# 1,3-Dichloroadamantyl-Containing Ureas as Potential Triple Inhibitors of Soluble Epoxide Hydrolase, p38 MAPK and c-Raf

**DOI:** 10.3390/ijms25010338

**Published:** 2023-12-26

**Authors:** Boris P. Gladkikh, Dmitry V. Danilov, Vladimir S. D’yachenko, Gennady M. Butov

**Affiliations:** 1 Department of Technology of Organic and Petrochemical Synthesis, Volgograd State Technical University, Volgograd 400005, Russia; gladkih-boris@mail.ru (B.P.G.); danilov.dmitry.vlz@yandex.ru (D.V.D.); gmbutov@mail.ru (G.M.B.); 2 Department of Chemistry, Technology and Equipment of Chemical Industry, Volzhsky Polytechnic Institute (Branch), Volgograd State Technical University (VSTU), Volzhsky 404121, Russia

**Keywords:** soluble epoxide hydrolase (sEH), cancer, p38 MAPK, inhibitors, signaling pathway, c-Raf, ureas

## Abstract

Soluble epoxide hydrolase (sEH) is an enzyme involved in the metabolism of bioactive lipid signaling molecules. sEH converts epoxyeicosatrienoic acids (EET) to virtually inactive dihydroxyeicosatrienoic acids (DHET). The first acids are “medicinal” molecules, the second increase the inflammatory infiltration of cells. Mitogen-activated protein kinases (p38 MAPKs) are key protein kinases involved in the production of inflammatory mediators, including tumor necrosis factor-α (TNF-α) and cyclooxygenase-2 (COX-2). p38 MAPK signaling plays an important role in the regulation of cellular processes, especially inflammation. The proto-oncogenic serine/threonine protein kinase Raf (c-Raf) is a major component of the mitogen-activated protein kinase (MAPK) pathway: ERK1/2 signaling. Normal cellular Raf genes can also mutate and become oncogenes, overloading the activity of MEK1/2 and ERK1/2. The development of multitarget inhibitors is a promising strategy for the treatment of socially dangerous diseases. We synthesized 1,3-disubstituted ureas and diureas containing a dichloroadamantyl moiety. The results of computational methods show that soluble epoxide hydrolase inhibitors can act on two more targets in different signaling pathways of mitogen-activated protein kinases p38 MAPK and c-Raf. The two chlorine atoms in the adamantyl moiety may provide additional Cl-π interactions in the active site of human sEH. Molecular dynamics studies have shown that the stability of ligand–protein complexes largely depends on the “spacer effect.” The compound containing a bridge between the chloroadamantyl fragment and the ureide group forms more stable ligand–protein complexes with sEH and p38 MAPK, which indicates a better conformational ability of the molecule in the active sites of these targets. In turn, a compound containing two chlorine atoms forms a more stable complex with c-Raf, probably due to the presence of additional halogen bonds of chlorine atoms with amino acid residues.

## 1. Introduction

### 1.1. Soluble Epoxide Hydrolase (sEH)

Soluble epoxide hydrolase (sEH) is an enzyme that hydrolyzes epoxides to the corresponding vicinal diols [1,2]. Mammalian sEH is mainly expressed in the cytosol [3], and its expression differs between animal species; for example, sEH is induced in rats than mice [4]. In addition, sEH is induced by peroxisome proliferator-activated α (PPARα) [3,5]. Inflammatory conditions are also the result of increased sEH expression [6]. A major place in the body’s response to an inflammatory response is the production of bioactive lipids from arachidonic acid (AA) [7]. Eicosanoids have protective effects and are vital AA metabolites in three metabolic pathways. The pathway in which soluble epoxide hydrolase (sEH) is involved is the cytochrome P450 (CYP) pathway, which forms EET and hydroxyeicosatetraenoic acids (HETE). EETs, in turn, are metabolized under the action of sEH to dihydroxyeicosatetraenoic acids (DHETs). The second and third pathways are the lipoxygenase (LOX) pathway, which catalyzes the formation of lipoxins and leukotrienes [8], and the cyclooxygenase (COX) pathway, which produces prostanoids [9]. Many AA metabolites along the cytochrome pathway are epoxides such as 8,9-, 11,12-, 14-15-EETs. 

The oxirane cycle is a strained reactive structure capable of reacting not only with proteins, but also with DNA, which leads to toxic and carcinogenic effects [10]. Study [11] shows that many signaling pathways play a role in EET-mediated angiogenesis, and one of them is the p38 MAPK pathway, which is activated by 8,9- and 11,12-EETs [12]. 

Both astrocyte-secreted EETs and synthetic EETs can stimulate endothelial cell proliferation, tube formation, and angiogenesis in the matrix gel in vivo [13,14,15]. Angiogenesis is critically dependent on endothelial cell migration [16]. EET has been shown to promote endothelial cell migration through eNOS, MEK/MAPK, and PI3-K [17]. It has also been shown that 11,12-EET can stimulate angiogenesis by activating the EGF receptor [18]. 14,15-EET has been shown to induce angiogenesis through several pathways, including Src, PI3K/Akt signaling in parallel with mTOR-S6K1 activation, and Src-dependent STAT-3 mediated VEGF expression [19,20]. The authors of [12] identified 5,6-EET and 8,9-EET as proangiogenic lipids. These regioisomers increased the density of blood vessels. This neovascularization has been enhanced by co-administration of a soluble epoxide hydrolase inhibitor that increases EET levels [12]. This study confirms the critical role of EET in angiogenesis. 

It can be concluded that sEH inhibition naturally produces an effect that manifests itself in a decrease in inflammatory conditions, but, in turn, “opens” new pathways leading to increased angiogenesis, as well as affecting growth factor receptors. A new strategy in therapy may be the study of inhibitors of soluble epoxide hydrolase sEH with high activity on other targets involved in inflammatory conditions and the progression of cancerous tumors.

In view of the fact that some EETs can activate EGF signaling receptors that activate p38 MAPK, it becomes obvious that the search for a strategy based on targeting potent sEH inhibitors to several more targets is an urgent and important task.

### 1.2. p38 MAPK

p38 proteins are a class of mitogen-activated protein kinases (MAPKs) that are major players during inflammatory responses, especially in macrophages. p38 expression is upregulated in response to inflammatory and stress stimuli such as cytokines, ultraviolet irradiation, osmotic shock, and heat shock and is involved in autophagy, apoptosis, and cell differentiation [21,22,23,24,25]. Accumulating evidence suggests that p38 plays an important role in inflammatory conditions in the liver, kidney, brain, and lungs, and that it plays an important role in macrophage-mediated inflammatory diseases [26,27,28]. TNF-α and IL-1 activate p38 isoforms by recruiting TRAF adapter proteins to the intracellular domains of their related receptors [29]. TRAF recruitment activates various MAPKKKs involved in the activation of p38 isoforms. The p38 isoforms are also activated by GPCRs [30], as well as by the Rho family GTPases Rac and Cdc42 [31]. It is believed that MKK3 and MKK6 are the main protein kinases responsible for the activation of p38 [32,33,34]. It is known that p38α is involved in the expression of pro-inflammatory mediators in macrophages, such as TNF-α, IL-1, IL-12 and PGE2 [35,36,37], as well as IL-8, IL-6, IL-3, IL-2 and COX-2 each of which contains AU-rich elements (ARE) in their three untranslated mRNA regions to which p38 binds [38]. It has also been reported that p38 can regulate the production of endothelial vascular cell adhesion molecule-1 (VCAM-1), which is involved in cell proliferation and immune response differentiation [39].

MKK3/6 activates p38 by means of double phosphorylation at the basic amino acid residues Thr180 and Tyr182, which are located in the activation loop in the Thr-Gly-Tyr motif [40].

Mitogen-activated protein kinases p38 is a promising target in the treatment of not only inflammatory conditions, but also cancer. 

In view of the fact that some EETs can activate VEGF signaling receptors that activate p38 MAPK, it becomes obvious that the search for a strategy based on targeting potent sEH inhibitors to several more targets is an urgent and important task.

### 1.3. c-Raf

Proto-oncogene serine-threonine protein kinase (c-Raf) is also known as c-Raf proto-oncogene or simply c-Raf (Raf-1). It is an enzyme [41] encoded in humans by the Raf1 gene [42,43]. The c-Raf protein is part of the ERK1/2 pathway in the form of MAP kinase (MAP3K), which functions downstream of the Ras subfamily of membrane-associated GTPases. 

There is a large amount of research on the activation of the RAS-RAF-MEK-ERK1/2 pathway [44,45,46,47]. This pathway is activated when many cell surface receptor tyrosine kinases in epidermal growth factor (EGFR) and platelet growth factor receptors (PGFR), as well as vascular endothelial growth factor receptors (VGFR), are activated by various stimuli, such as cytokines, mitogenic factors, etc. [48,49,50]. The receptors begin to attract adapter proteins (GBR2) associated with growth receptors, which then bind to guanine nucleotide exchange factor Son of Sevenless (SOS). Further, SOS, located close to the RAS GTPase associated with GTP, induces conformational changes in the RAS and replaces GDP with GTP, thereby bringing it into an active state [51]. Further, GDP activates the RAF family of kinases by means of hetero- or homodimerization [52], and RAF, in turn, activates MEK1/2 and the latter finally activates ERK1/2 [53,54,55,56,57,58].

### 1.4. Signaling Pathways

**Figure 1:**
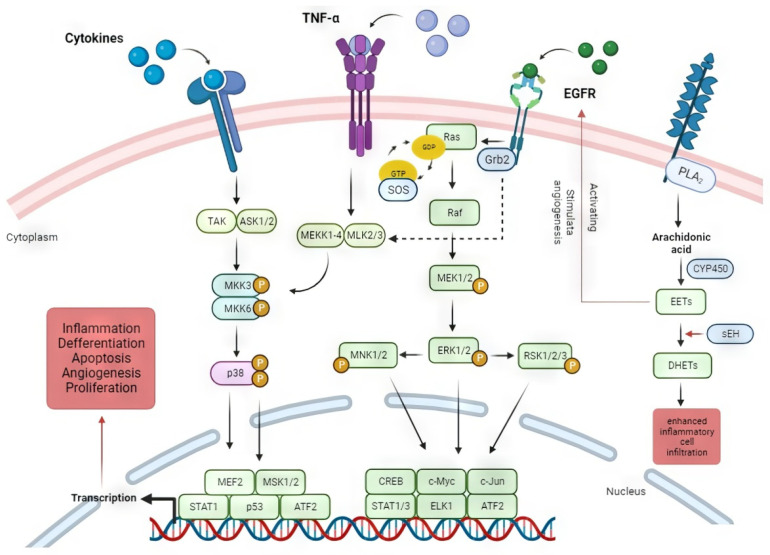
Signaling pathways that unite the described targets.

The ERK1/2 pathway is one of the major cellular signaling pathways that combines multiple extracellular signals with the corresponding cellular responses by phosphorylation and induction of multiple downstream targets, including c-Jun, ATF-2, Elk-1, RSK1–3, MNK1/2, and others (Figure 1). Through its kinase activity, it can regulate cell differentiation, cell cycle progression, apoptosis, gene expression, proliferation, invasiveness, and metastasis [59,60,61]. Figure 1 also shows the p38 MAPK activation pathway by cytokines and TNF-α, which initiates the production of pro-apoptotic transcription factors for inflammation, differentiation, proliferation, and apoptosis. Also described is the conversion of AA to EETs and DHETs under the action of enzymes CYP and sEH. EETs activate EGFR, which leads to stimulation of angiogenesis.

The ERK1/2 pathway is the most frequently deregulated kinase pathway in approximately 30% of human cancers, largely because the upstream MEK1/2 activator genes, namely RAS and RAF, undergo mutations that render the respective proteins independent of upstream activating signals or unable to undergo deactivation [62,63,64,65]. Increasingly, new mutations are being identified in cancer patients [66,67,68]. Significant progress has recently been achieved in the successful development of targeted therapies against key players along the way: pan-RAF inhibitor sorafenib, BRAFV600E (oncogenically mutated BRAF) inhibitors vemurafenib and dabrafenib, and the MEK1/2 inhibitor trametinib for monotherapy [69,70]. The development of inhibitors for RAS, one of the most frequently mutated oncogenes, is more challenging as none of the available inhibitors are approved for routine treatment.

It is worth saying that the ab initio method of quantum chemistry is widely used in the study of biological molecules. For example, based on this method, dynamic functional density modeling of ssRNA interacting with Na^+^ or Mg^2+^ countercations in aqueous solution identified high-affinity binding sites that coordinated metal ions for extended periods of time exceeding 100 ps [71]. Also, the authors in their other works [72] used the DFT method with the help of which various methanol complexes with hydrogen bonds with various proton–acceptor and proton–donor molecules containing functional groups Cl, F, NH 2, OH, OR and COOH were modeled. The DFT method is often used to study biological systems, but apparently with smaller models.

Based on the above data, a simple conclusion can be drawn that the search and development of inhibitors that target not one enzyme, but two or more targets, that is, that are multitargeted, that terminate the signaling pathways responsible for inflammatory reactions, tumor conditions, and proliferation of mutant cells, is a promising strategy.

## 2. Inhibitors of sEH, p38 MAPK and c-Raf

**Figure 2:**
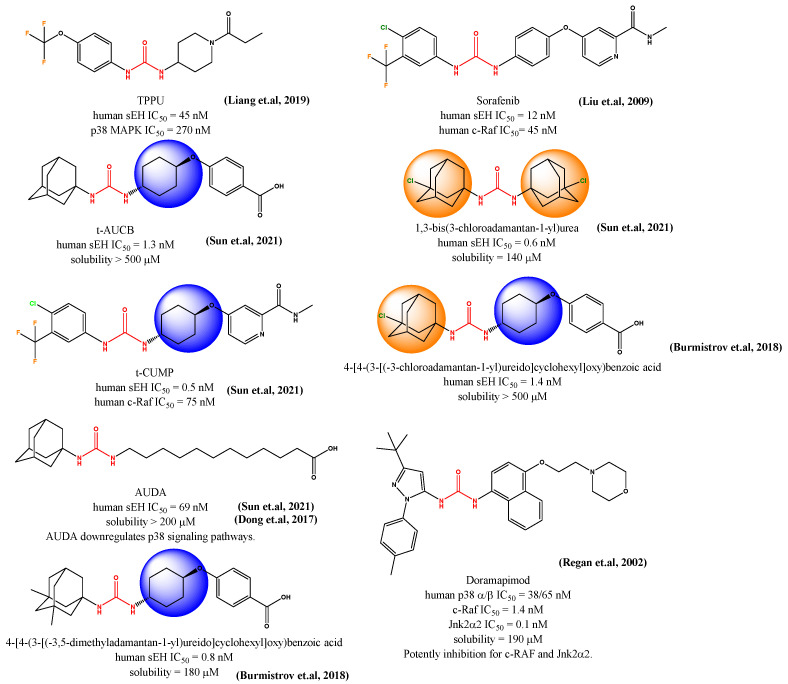
Known inhibitors with activity against soluble epoxide hydrolase sEH, p38 MAPK and c-Raf [73,74,77,78,79,80].

The authors report [73] the activity of 1-Trifluoromethoxyphenyl-3-(1-propionylpiperidin-4-yl) urea (TPPU) as a potent dual inhibitor of soluble epoxide hydrolase sEH and p38 MAPK. In work [74], the inhibitory activity of sorafenib against sEH was studied. Sorafenib has the same pharmacophore center as known sEH inhibitors (Figure 2). Sorafenib is a well-known drug—a low-molecular-weight multikinase inhibitor that inhibits not only intracellular kinases (serine/threonine kinases c-Raf, BRaf and mutant BRaf), but also receptor tyrosine kinases located on the cell surface such as vascular endothelial growth factor receptors (VEGFR-1, VEGFR-2 and VEGFR-3), platelet growth factor receptor (PDGFR-β) and neurotrophic glial factor receptor (RET). Sorafenib treatment induces autophagy [75] which may suppress tumor growth. Based on its 1,3-disubstituted urea structure, sorafenib is also a potent soluble epoxide hydrolase inhibitor, and this activity likely reduces the severity of its adverse effects [76].

In this work, 1,3-dichloroadamantyl-containing ureas and diureas are synthesized. As can be seen, one chlorine atom in the nodal position of the adamantyl fragment does not significantly change the inhibitory activity against soluble epoxide hydrolase sEH IC_50_ = 1.4 nM (Figure 2).

Work [78] shows that the microsomal stability of ureas depends on the lipophilicity of the adamantyl substituent. The introduction of methyl groups into the adamantine framework reduces stability from ~60 min to ~5 min (for trisubstituted adamantane). It has also been shown that the activity of urea containing a 1,3-dimethyladamantyl fragment on the left side of the molecule and trans-4-amino-(cyclohexyloxy)benzoic acid on the right side has an IC_50_ of 0.8 nM (Figure 2).

Due to the fact that the van der Waals radii of the chlorine atom and the methyl group are close in value (2.0 Å and 1.8 Å, respectively), it can be assumed that chlorine has the same positive effect as methyl substituents when it comes to biochemical activity and, in turn, without reducing microsomal stability.

Chlorine can also improve lipophilic binding and form halogen bonds. A new study [81] suggests that such bonds may be possible. The work states that there is a positive electrostatic potential (σ-hole) at the end of the chlorine atom. This double charge allows for the chlorine substituent to act as a “head-to-head” halogen bond donor. The fluorine atom does not have a positively charged region due to its greater electronegativity, and therefore cannot interact with nucleophiles. Thus, chlorine-containing molecules have more possible ways of binding and may be much more effective than fluorine-containing molecules, and at least to the same extent as molecules containing methyl substituents. 

In this regard, we choose the 1,3-dichloro-adamantyl fragment as a motif for the creation of potential inhibitors.

Adamantane is a highly lipophilic moiety that has the ability to increase the solubility of the molecule as a whole. In addition, adamantane scaffolds improve the metabolic stability, pharmacokinetics and membrane transport of modified drugs [81,82,83,84].

The adamantane moiety is capable of providing drug stability, leading to improved pharmacokinetics of modified drug candidates [85,86]. The rigid scaffold fragment protects nearby functional groups from metabolic degradation, which leads to increased stability and distribution of the drug in the blood plasma [87]. Adamantane can also be incorporated into the lipophilic portion of the lipid bilayer that makes up membranes [88], which is an important first step for drug transport across cell membranes.

The introduction of two chlorine atoms further increases the lipophilicity of the adamanatane framework and the compound molecule as a whole. Increasing the lipophilicity of the entire molecule by chlorine atoms results in a higher distribution of the chlorinated compound into the lipophilic phase of the cell membrane or lipophilic domains of the protein. This causes a higher local concentration of the compound near the biological target point, but not necessarily higher biological activity. The properties mentioned above result in steric and electronic effects of chlorine substituents and local electronic attraction or repulsion or steric interaction with any amino acid residue surrounding the chlorine atom position in the binding pocket of the protein. This, in turn, can cause closer interaction or weakening of contacts with amino acids close to chlorine or in other parts of the active molecule. Any of these can affect the function of the target protein and cause an increase or decrease in biological activity. The ability of the chlorine atom to form weak bonds with lipid molecules, as well as the ability to electrostatically attract electrons to itself, forming temporary dipoles and creating positive charges with lipid molecules, leads to improved affinity with the lipid layer and better ability to integrate into lipid structures, which leads to better permeability through the cell membrane.

It is worth paying attention to the given data on the water solubility of compounds. Poor solubility and low permeability are the most common barriers to oral bioavailability in drug discovery. The solubility of a drug candidate has one of the greatest influences on the desired concentration of the drug in the systemic circulation and, therefore, on whether the drug produces the desired effect or pharmacological response in patients. Figure 2 shows water solubility data. It can be seen that 4-[4-(3-[(-3,5-dimethyladamantan-1-yl)ureido]cyclohexyl]oxy)benzoic acid and 4-[4-(3-[(-3-chloroadamantan-1-yl)ureido]cyclohexyl]oxy)benzoic acid have very similar inhibitory activity values (0.8 and 1.4 nM), but their water solubility is very different (180 and >500 μM). The introduction of chlorine atoms into the adamantyl fragment can increase the water solubility of inhibitors without losing their activity.

The 1,3-dichloroadamantyl fragment can be an excellent structural motif for improving the physicochemical and biochemical properties of the synthesized compounds.

## 3. Results and Discussion

### 3.1. Synthesis and Methods

The literature describes [89] methods for the synthesis of 3,5-dichloroadamantylcarboxylic acid **4**, which can act as a precursor for the production of isocyanate **5**. The literature describes a method for the synthesis of acid **4** from adamant-1-yl carboxylic acid with a yield of 84%, which consists in using tetrachloride carbon as a chlorinating agent, and magnesium triacetyl acetonate as a catalyst. The reaction is carried out in a microautoclave at a temperature of 200 °C.

**Scheme 1:**
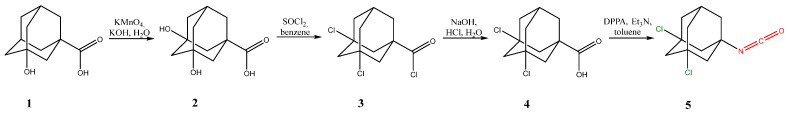
Preparation of 3,5-dichloroadamant-1-yl isocyanate.

To obtain 3,5-dihydroxyadamantan-1-yl carboxylic acid **2** as a precursor for obtaining dichlorocarboxylic acid **4**, 3-hydroxyadamantan-1-yl carboxylic acid **1** was reacted with permanganate and potassium hydroxide in an aqueous solution at the boiling temperature of the solvent for 5 h (Scheme 1). The product yield after purification was 37%. The total yield was 82%.

The resulting 3,5-dihydroxyadamantan-1-yl carboxylic acid **2** was treated with a 3.3-fold molar excess of thionyl chloride in dried benzene. The reaction was carried out at room temperature for 24 h. The reaction mixture was concentrated to produce acid chloride **3**. The yield of acid chloride intermediate **3** was 91%.

Further, the resulting acid chloride **3**, after removal of the solvent, was subjected to alkaline hydrolysis at room temperature for 8 h. The addition of HCl led to the formation of 3,5-dichloroadamantan-1-yl carboxylic acid **4** in 68% yield.

By treating 3,5-dichloroadamantan-1-yl carboxylic acid **4** with equimolar amounts of diphenylphosphoryl azide (DPPA) and triethylamine, 3,5-dichloroadamantan-1-yl isocyanate 5 was synthesized in 88% yield.

**Scheme 2:**
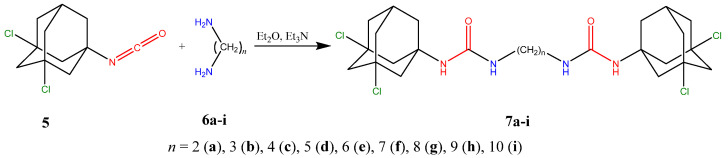
Preparation of 1,3-dichloroadamantyl containing diureas **7a**–**i**.

**Scheme 3:**
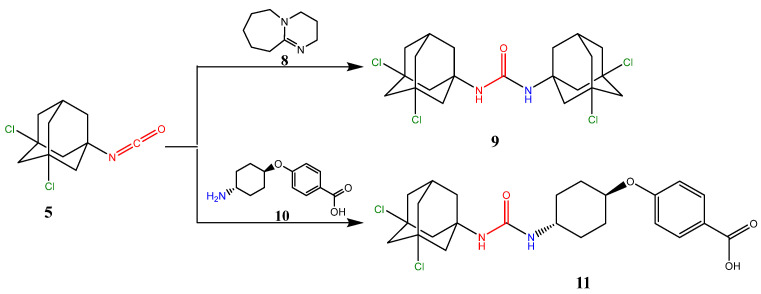
Preparation of symmetrical 1,3-disubstituted urea **9** and **11**.

Based on 3,5-dichloroadamantan-1-yl isocyanate **5**, symmetrical diureas **7a**–**i** (Scheme 2) and ureas **9** and **11** (Scheme 3) containing a 3,5-dichloroadamantyl moiety were obtained for the first time.

For the synthesis of 1,3-disubstituted ureas and diureas, aliphatic diamines were chosen (Scheme 2), from which the most active inhibitors of human soluble epoxide hydrolase (hsEH) were previously obtained [90].

The synthesis of disubstituted diureas and ureas was carried out in anhydrous diethyl ether for 12 h at room temperature in the presence of an equimolar amount of triethylamine.

For the synthesis of 1,3-disubstituted urea **11**, trans-4-amino-(cyclohexyloxy)benzoic acid **10** was used, from which the most active inhibitors of soluble epoxide hydrolase (hsEH) were previously obtained (t-AUCB, c-AUCB, t-chloroAUCB) [78].

Symmetrical 1,3-disubstituted urea **9** was obtained by the reaction of 3,5-dichloroadamantan-1-yl isocyanate **5** with water in THF and in the presence of 1,8-diazabicyclo[5.4.0]undec-7-ene (DBU) (Scheme 3).

In the ^1^H NMR spectra of compounds **7a**–**i**, obtained from (3,5-dichloroadamantan-1-yl)isocyanate **5**, the chemical shift of ^1^NH protons is in the range of 5.68–6.24 ppm, and the signals of ^3^NH protons associated with the dichloroadamantyl fragment shifts to a weaker field of 5.85–7.26 ppm. In the ^1^H NMR spectra of compound **9**, the NH proton signals are located in the region of 5.90 ppm, and the signal of the ^1^NH protons of compound **11** is located in the region of 5.74 ppm, and the signal of ^3^NH protons is in the region of 5.88 ppm.

The cLogP values increase depending on the increase in the number of bridges in the corresponding diureas (Table 1). Diurea **7a** (5.74) is already beyond the Lipinski rule. The highest indicator is for symmetrical urea **9**. Compound **11**, which is chosen as a compound for computer calculations, has the lowest cLogP value (4.81).

The melting points of ureas **7a**–**i**, **9** and **11** are in the range of 76–230 °C (Table 1). Two chlorine atoms in the nodal positions of the adamantyl fragment increase the melting point of symmetrical urea by almost 100 °C compared to similar urea containing one chlorine atom in adamantane (132 °C) [78]. However, in regard to urea **11**, the presence of two chlorine atoms in adamantane reduces the melting point of the compound by 34 °C compared to the analogous monochlorourea (243 °C) [78]. Diureas **7d** and **7e** have the lowest melting points. Compared to the same ureas containing one chlorine atom in the adamantyl radical, two chlorine atoms reduce the melting points by 44 °C and 29 °C, respectively [78].

### 3.2. Molecular Docking and MD Simulation

Molecular docking of ligands was carried out against three targets—soluble epoxide hydrolase (sEH), p38 MAPK and c-Raf.

#### 3.2.1. Soluble Epoxide Hydrolase (sEH)

In view of the fact that the known ureas containing the trans-4-amino-(cyclohexyloxy)benzoic acid moiety exhibit potent inhibitory activity against soluble epoxide hydrolase sEH, it was decided to consider putative modes of binding 4-[4-((3-[(-3-chloroadamantan-1-yl)methyl)ureido]cyclohexyl]oxy)benzoic acid (**12**) and **11** with sEH by molecular docking and MD simulation. Compound **12** was chosen as an analogue of compound **11**, containing a spacer between the adamantyl moiety and the ureide group.

Molecular docking was performed with complexes **11** and **12** with sEH (PDB: ID: 5AM3).

**Figure 3:**
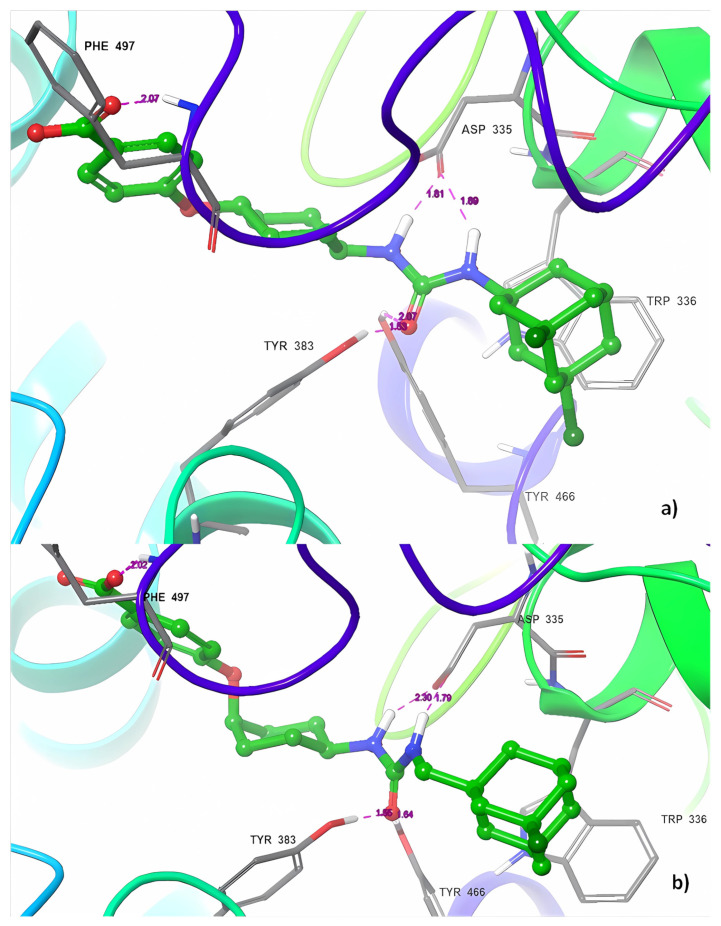
(**a**) Molecular docking of **11** with sEH. MM/GBSA binding free energy −56.52 kcal/mol. (**b**) Molecular docking of **12** with sEH. MM/GBSA binding free energy −57.26 kcal/mol. Purple dotted lines show the distance between atoms.

The urea fragment of ligand **11** forms four hydrogen bonds in the active pocket of the enzyme with amino acid residues Tyr383, Tyr466 and Asp335 (Figure 3). The adamantane fragment is located inside the hydrophobic pocket formed by Trp336, Met 339, Ile363, and Phe381 residues. Also, the carbonyl atom of the acid group is capable of forming an additional hydrogen bond with the amino acid residue Phe497. MM/GBSA binding free energy is −56.52 kcal/mol. It should be noted that the 1,3-dichloroadamantyl fragment is located near the aromatic amino acid residues Trp336 and Phe381. The chlorine atom near Trp336 is located at a distance of 3.3 Å, the second chlorine atom near Phe381 is located at a distance of 3.18 Å. Due to the fact that there is no spacer between the urea fragment and the adamantyl radical, adamantane is not capable of “rotation” or “bending”. This makes it possible for the chlorine atoms to form an interaction of Cl-π with Trp336 and Phe381 “edge on” with the energy of 2.01 kcal/mol and the main source of attraction in the form of dispersion forces [91].

Compound **12** forms the same hydrogen bonds as compound **11** in a complex with sEH. The binding free energy is slightly better than that of **11**. It was worth comparing the molecular dynamics simulations of these complexes to see the differences in interactions.

**Figure 4:**
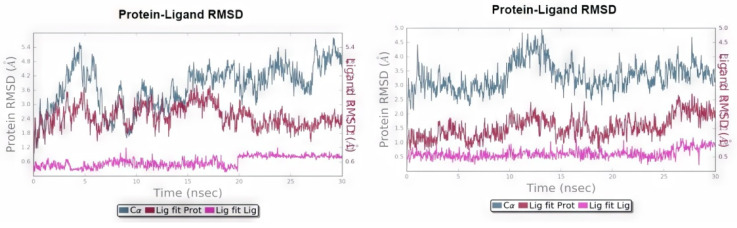
RMSD of **11** and **12** with sEH.

It can be seen that complex **12** with sEH is more stable throughout the entire simulation time than complex **11** with sEH and has fewer deviations (Figure 4). A simulation time of 30 ns was chosen to compare the stability of the complexes.

**Figure 5:**
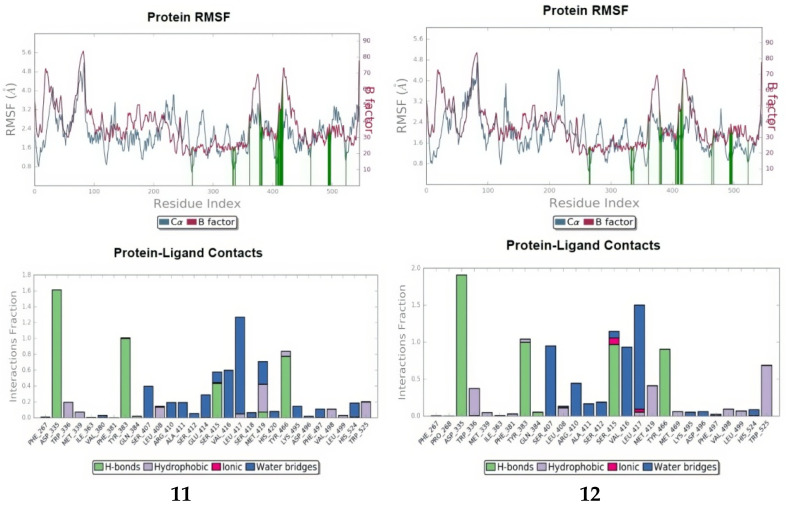
RMSF of **11** and **12** and protein-ligand contacts with sEH.

Complexes **11** and **12** with sEH have almost identical fluctuations, as can be seen from the RMSF plots. We can say that the interactions of the ligands with the protein are the same; however, complex **12** with sEH shows better interaction with Asp335, as well as a longer contact time with Ser415 (Figure 5).

**Figure 6:**
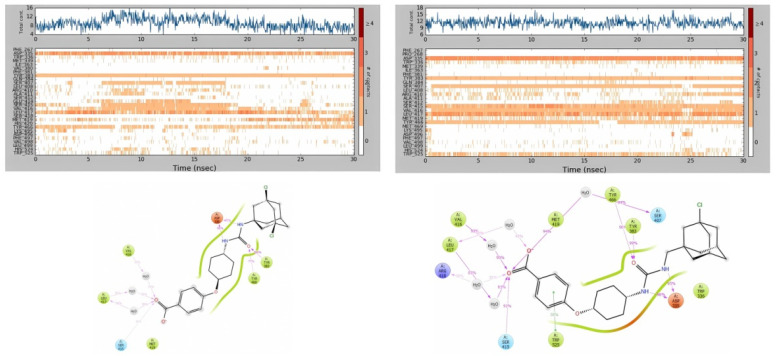
Protein–ligand contacts of **11** and **12** with sEH.

It can be seen that complex **12** with sEH has better and more stable interactions than complex **11** with sEH (Figure 6). Complex **12** with sEH exhibits stable interaction with Ser407 throughout the simulation (94% of the “time”), as well as better interaction with Ser415 (92% of the “time”). This may be due to the more flexible structure of **12** compared to **11** due to the spacer between the adamantyl radical and the ureide group, which offers additional rotations and a more flexible conformation.

The synthesized compounds may have potent activity against the soluble epoxide hydrolase sEH. However, it is worth considering introducing a bridge between the adamantyl moiety and the ureide group to provide additional opportunity for rotation of the molecule in the active site of the enzyme. The two chlorine atoms in the adamantane molecule provide additional interactions with the aromatic rings of amino acids Trp336 and Phe381. It is possible that this can affect the inhibitory activity of the synthesized compounds, although the content of two chlorine atoms in the adamantane molecule is likely to have a greater impact on the metabolic stability of the compounds.

#### 3.2.2. p38 MAPK

The p38 MAP kinase plays a critical role in regulating the production of pro-inflammatory cytokines such as TNFa, IL-1, and many others. Blocking this kinase may offer effective therapy for many inflammatory diseases. Article [92] reports a novel allosteric binding site for a class of ureas that are potent and selective inhibitors of human p38 MAP kinase. The formation of the binding site occurs with large conformational changes not previously identified for any of the Ser/Thr protein kinases. This change occurs in the highly conserved Asp-Phe-Gly motif (DFG motif) at the active site of the kinase. The authors of the study argue that a class of compounds such as ureas have slow binding kinetics, which is consistent with the requirement of conformational change. BIRB 796 (Doramapimod) is a well-known p38 MAPK inhibitor of the urea class [80]. Doramapimod is only similar in pharmacophore group (urea) to our synthesized compounds, so it makes no sense to compare binding free energies due to strong structural differences.

Molecular docking was performed with complexes **11** and **12** with p38 (PDB ID: 1KV1, PDB ID: 3HEG).

**Figure 7:**
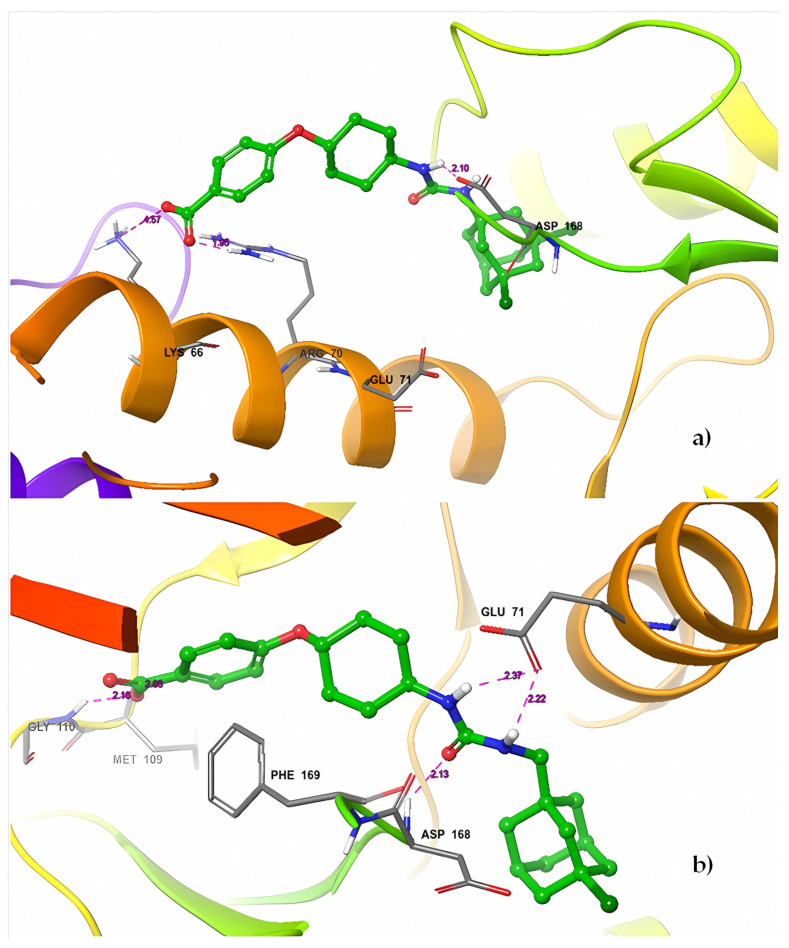
(**a**) Molecular docking of **11** with p38 MAPK (1KV1). MM/GBSA binding free energy −23.63 kcal/mol. (**b**) Molecular docking of **12** with p38 MAPK (1KV1). MM/GBSA binding free energy −35.34 kcal/mol. Purple dotted lines show the distance between atoms.

Figure 7 shows that the urea group forms only one hydrogen bond with the Asp168 amino acid residue from the DFG motif. It can be seen that two protons of *NH*-groups are far removed from the Glu71 amino acid residue, which, according to study [93], is involved in the conformational changes in the ATP-binding pocket. However, the carbonyl atom of the acid forms an additional hydrogen bond with the Arg70 residue, which can offer gain in the stability of this complex. The carboxyl group also has a very weak salt bridge with Lys66, with a distance of 4.57 Å.

The presence of two chlorine atoms in adamantane apparently creates steric difficulties for all possible “rotations” of the molecule, which leads to an “unfortunate” location of the inhibitor in the active center.

It is also worth mentioning that the conformationally rigid structure of the adamantyl fragment, combined with the absence of a spacer between the urea group and adamantane, makes it impossible to “bend” the chain and change the conformation of the inhibitor molecule as a whole.

To verify this statement, we calculated the molecular docking of the **12** molecule, which has a bridge between the urea group and adamantane.

As can be seen, the inhibitor molecule occupies the ATP-binding pocket, forming two hydrogen bonds with the Glu71 amino acid residue, and also interacts with the front end of the DPG loop, forming a hydrogen bond with the Asp168 residue. In addition, the carboxyl group of the inhibitor enters the allosteric pocket of the enzyme, forming hydrogen bonds with the amino acid residues Met109 and Gly110. MM/GBSA binding free energy is −35.34 kcal/mol.

Molecular dynamics simulation calculations were also performed.

**Figure 8:**
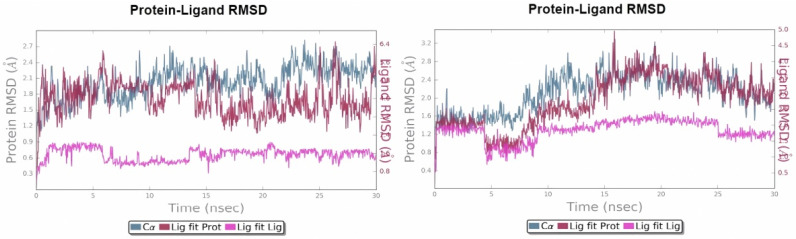
RMSD of **11** and **12** with p38 MAPK (PDB ID: 1KV1).

It can be seen that **12** forms a more stable complex with the allosteric binding site of p38 MAPK (Figure 8). The primary deviation may be due to sudden heating of the system and the addition of ions. Throughout the entire simulation, the deviation does not go beyond 2Å, which indicates a more stable complex than in the case of **11**.

**Figure 9:**
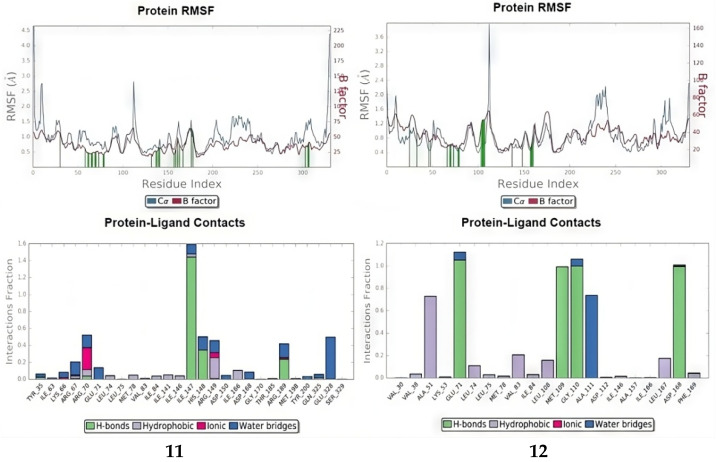
RMSF of **11** and **12** and protein–ligand contacts with p38 MAPK (PDB ID: 1KV1).

It can be seen from the figure that in the case of both complexes, the fluctuations are almost identical. However, in case **12**, we see more stable and long-lasting interactions with amino acid residues, which may play a significant role in the formation of a stable lignad–protein complex (Figure 9). Also, a more stable interaction of **12** is indicated by a long time of contact with amino acid residues.

**Figure 10:**
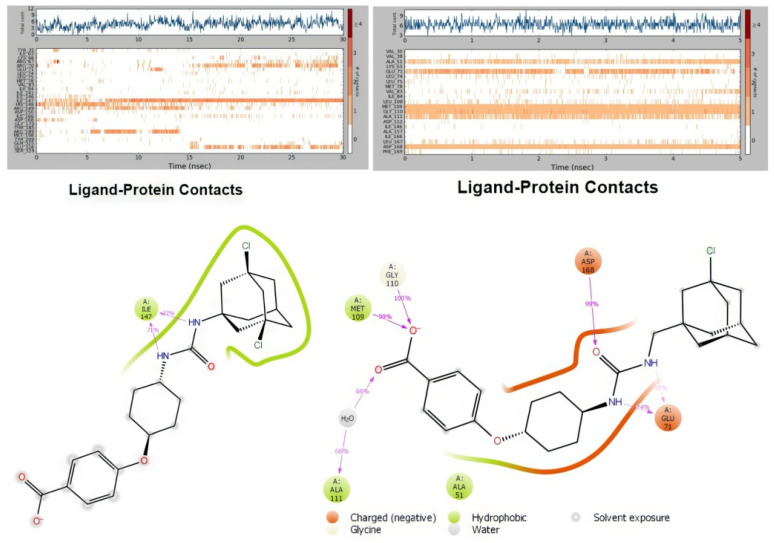
Protein–ligand contacts of **11** and **12** with p38 MAPK (PDB ID: 1KV1).

Urea group 11 forms only two stable hydrogen bonds with Ile147 70% of the time. In contrast, the carbonyl on urea group of **12** forms a stable contact with Asp168 for almost 100% of the simulation time (Figure 10). Also, two *NH*-groups form contacts with Glu71. The carboxyl group is also involved in the formation of very stable hydrogen bonds (almost 100% of the contact time) with Met109 and Gly110. These stable interactions **12** are likely due to conformational mobility associated with the presence of a spacer between the adamantyl moiety and the urea group.

A small conclusion can be drawn that the “spacer effect” makes a significant contribution to the conformational changes in the inhibitor molecule and allows the formation of a more stable enzyme–inhibitor complex.

We also considered the possibility of inhibiting the active site of p38 MAPK (PDB ID: 3HEG).

**Figure 11:**
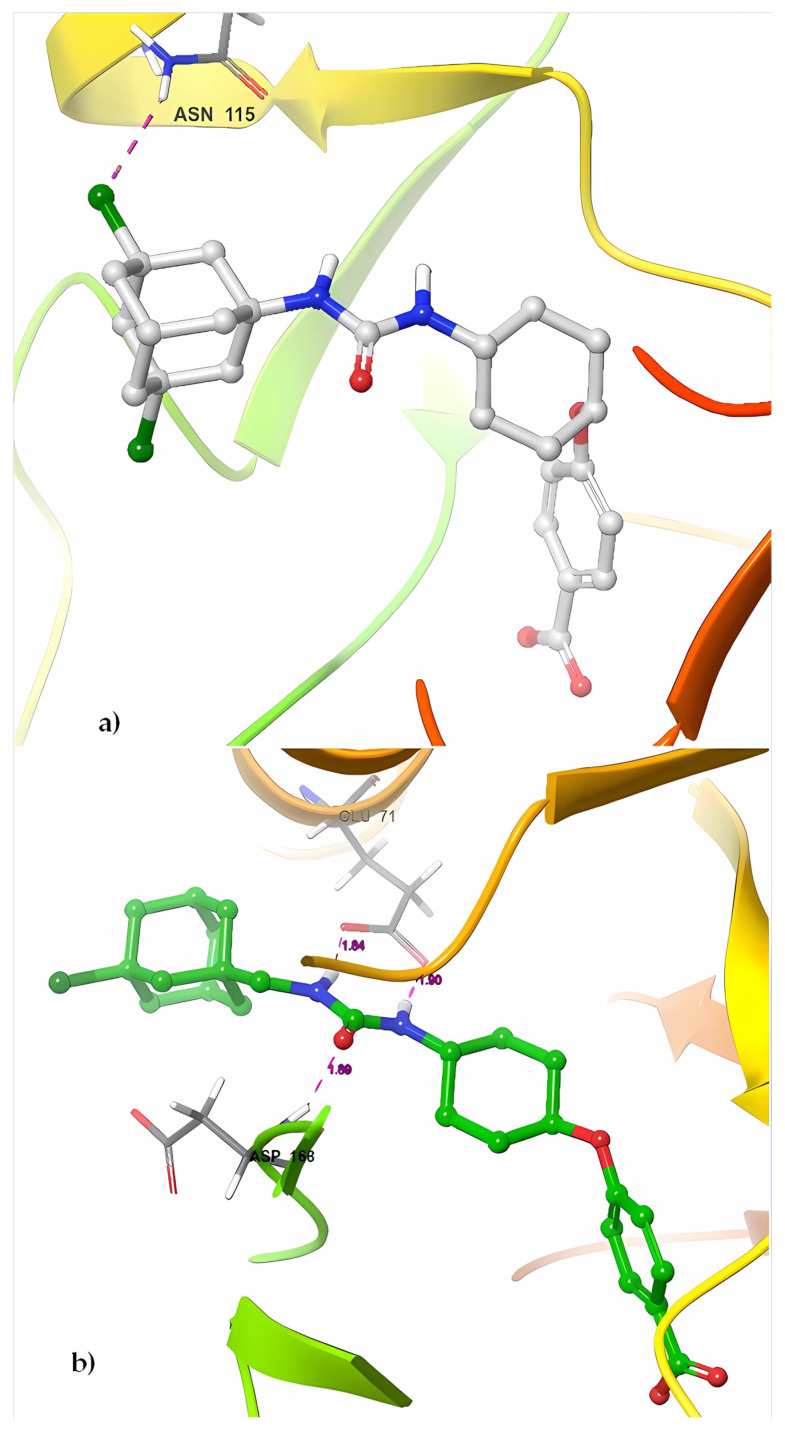
(**a**) Molecular docking of **11** with p38 MAPK (PDB ID: 3HEG). MM/GBSA binding free energy −27.60 kcal/mol. (**b**) Molecular docking of **12** with p38 MAPK (PDB ID: 3HEG). MM/GBSA binding free energy −26.33 kcal/mol. Purple dotted lines show the distance between atoms.

From the molecular docking data, it is already clear that **11** is poorly located in the active site of the enzyme, forming only one halogen bond with Asn115. If we compare it with **12**, the latter occupies a more favorable position, as can be seen from molecular docking (Figure 11).

The molecule forms three hydrogen bonds with residues Asp168 and Glu71, but there is no halogen bond due to the absence of an additional chlorine atom in the adamantyl fragment. Molecular dynamics simulations should provide greater clarity.

**Figure 12:**
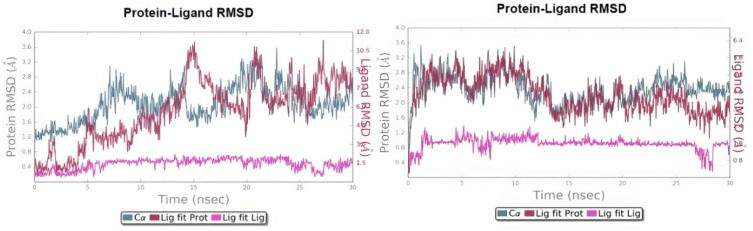
RMSD of **11** and **12** with p38 MAPK (PDB ID: 3HEG).

Both complexes have significant deviations, although complex **12** with p38 MAPK has less deviations than complex **11** with p38 (Figure 12). It is likely that even compound **11** leaves the active site of the enzyme.

**Figure 13:**
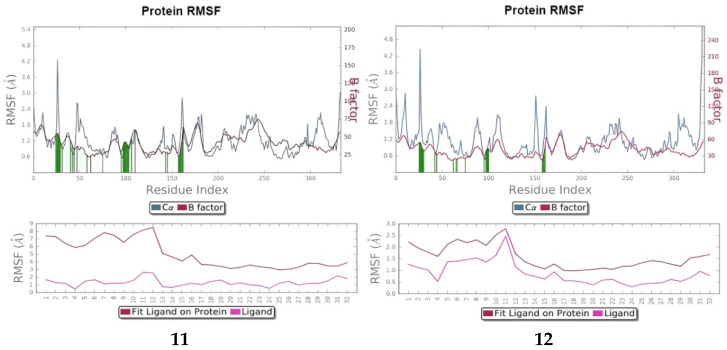
RMSF of **11** and **12** with p38 MAPK (PDB ID: 3HEG).

From the RMSF data, it is clear that protein fluctuations are almost the same. However, if the fluctuations of **11** on the protein are examined, it can be seen that almost all the atoms of the molecule are beyond 4 Å, which cannot be said about molecule **12**, in which all atoms fluctuate within 2.5 Å (Figure 13). This may also be evidence that “spacer effect” contributes to the conformational stability of the molecule. Considering the fact that the adamantane framework is a very rigid structure, the presence of such a bridge makes it possible to avoid steric hindrances in the active site of the protein due to bending of the molecule.

**Figure 14:**
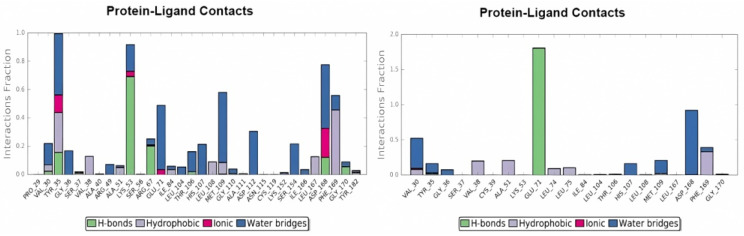
Diagram of protein–ligand contacts of **11** and **12** with p38 MAPK (PDB ID: 3HEG).

**Figure 15:**
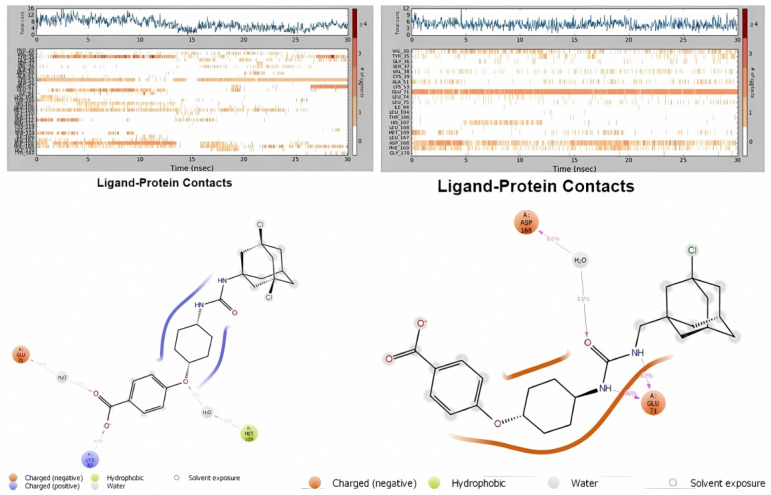
Protein–ligand contacts of **11** and **12** with p38 MAPK (PDB ID: 3HEG).

From Figure 14, it may seem that **11** forms quite a lot of contacts with the protein, but all of them are short-lived and not stable enough, as Figure 15 shows.

The urea group **11** does not form any contacts throughout the simulation, only short-term interactions of the carboxyl group (Figure 15). On the contrary, if **12** is examined, it can be seen that the urea group is involved in interactions with Glu71 and Asp168. Again, the bridge between the adamantyl moiety and the urea group facilitates better folding of the molecule into the active site.

It cannot be said that these compounds are active against these sites of p38 MAPK; however, it is worth noting that when developing new inhibitors targeting p38, this knowledge can be used to obtain molecules with convenient conformational characteristics.

Perhaps, it is worth considering the creation of an inhibitor containing a 1,3-dichloroadamantyl fragment and a spacer between the urea group and adamantane.

#### 3.2.3. c-Raf

The human c-Raf gene is located on chromosome 3. Like many other MAPKKKs, c-Raf is a multidomain protein with several additional domains to help regulate its catalytic activity. At its N-terminal region, the Ras-binding domain (RBD) and the homologous C-kinase domain 1 (C1-domain) are located next to each other.

The Ras-binding domain has a ubiquitin-like fold (like many other small G-protein-associated domains) and selectively binds only GTP-bound Ras proteins [94,95,96].

The C1 domain, just downstream of the Ras-binding domain, is a distinct zinc finger that is rich in cysteines and stabilized by two zinc ions. It is similar to the diacylglycerol-binding C1 domains of protein kinase C (PKC) enzymes [97,98]. But unlike PKC, the C1 domains of Raf family kinases do not bind diacylglycerol [99]. Instead, they interact with other lipids such as ceramide [99] or phosphatidic acid [100] and even help recognize activated Ras (GTP-Ras) [101].

The close proximity of these two domains, as well as a number of experimental data, suggest that they act as a single unit, negatively regulating the activity of the protein kinase domain through direct physical interaction [102]. Historically, this autoinhibitory block was labeled as the CR1 region (“conserved region 1”), the hinge region was called CR2, and the kinase domain was called CR3. Unfortunately, the exact structure of the autoinhibited kinase remains unknown.

Between the autoinhibitory domain block and the catalytic kinase domain, one can find a long segment that is characteristic of all Raf proteins. It is highly enriched in serine amino acids, but its exact sequence is poorly conserved in related Raf genes. This area seems inherently unstructured and very flexible. Its most likely role is to act as a natural “hinge” between tightly folded autoinhibitory and catalytic domains, enabling complex movements and deep conformational rearrangements within the molecule [103].

And yet, the main domain where the binding of inhibitors occurs is the protein kinase domain, which contains the N-terminal and C-terminal lobes. In c-Raf, these are amino acid residues from 350 to 620.

Molecular docking was performed with complexes **11** and **12** with c-Raf (PDB: ID: 3OMV).

**Figure 16:**
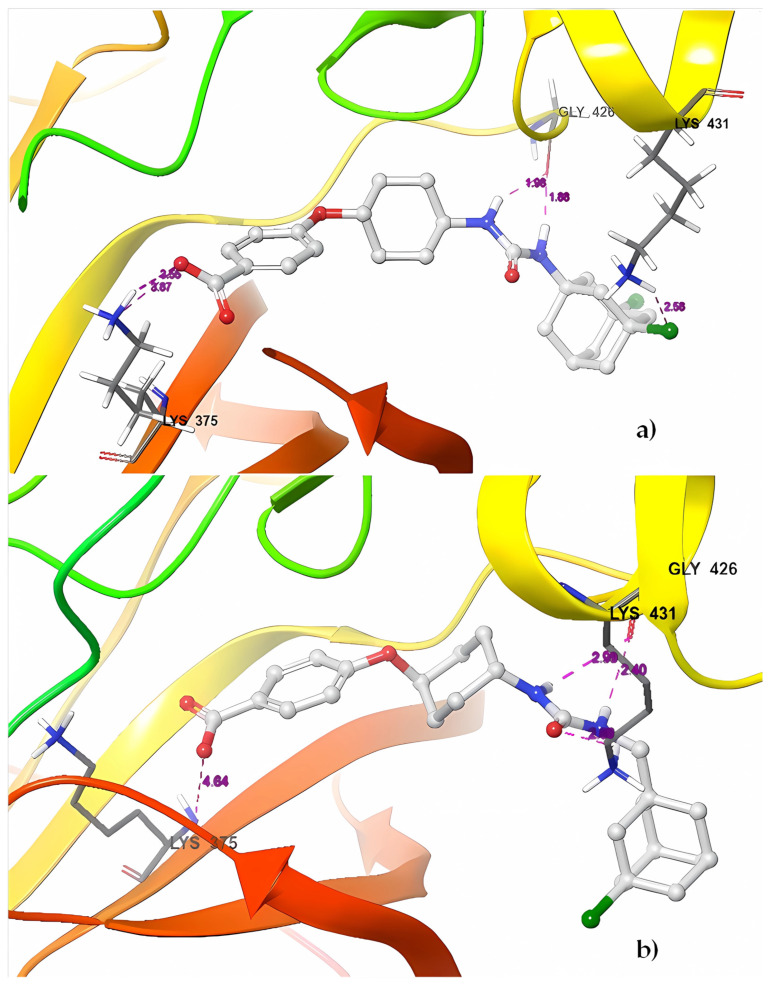
(**a**) Molecular docking of **11** with c-Raf. MM/GBSA binding free energy −29.78 kcal/mol. (**b**) Molecular docking of **12** with c-Raf. MM/GBSA binding free energy −32.75 kcal/mol. Purple dotted lines show the distance between atoms.

As can be seen from Figure 16, the urea fragment of compound **11** forms two hydrogen bonds with the Gly426 amino acid residue. The carboxyl group forms a salt bridge with the Lys375 amino acid residue (distance 3.37 Å). Chlorine atoms do not participate in the interaction of Cl-π, since aromatic rings of amino acid residues are not located nearby. It can be seen that there is a formation of a halogen with one of the chlorine atoms with Lys431.

It is possible that the presence of two chlorine atoms in the adamantane molecule adversely affects the conformational abilities of the inhibitor molecule as a whole, which in turn affects the free energy of binding. Also, it should be noted that carbonyl oxygen does not form hydrogen bonds.

Therefore, it was decided to calculate the free energy of binding of an inhibitor molecule containing one chlorine atom in the nodal position of the adamantyl radical.

In view of the fact that, when calculating the binding energies of p38 MAPK, we saw a “spacer effect” on the free energy of binding compounds, it was worth also calculating **12**.

Figure 16 shows that the urea fragment forms three hydrogen bonds with amino acid residues Gly426 and Lys431, but as in the case of **11**, one of the bonds has an even weaker interaction (distance 2.99 Å). The carboxyl fragment is also unable to form salt bridges with Arg or Lys residues due to the distance of more than 4.5 Å.

Although **12** has a more negative MM/GBSA binding free energy, its hydrogen bonds may be less strong than those of compound **11**, which forms two hydrogen bonds with the Gly426 residue (distances 1.88 and 1.96 Å, Figure 16). However, it is worth considering this in dynamics.

**Figure 17:**
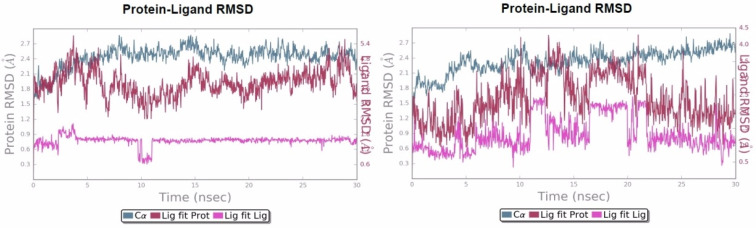
RMSD of **11** and **12** with c-Raf.

Both complexes likely extend beyond the protein’s active site. It is clear that **12** is trying to find a more favorable conformational position and adapt to the active center, so fluctuations are visible (Figure 17). However, this may be due to insufficient simulation time for this particular system.

**Figure 18:**
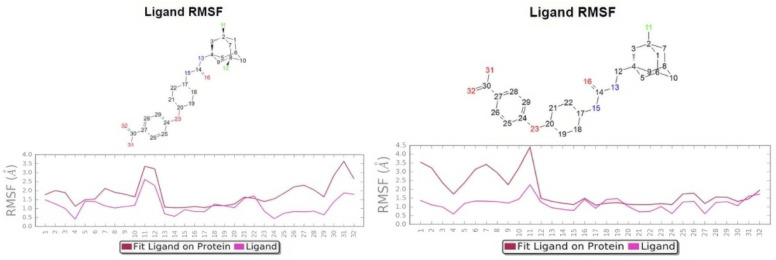
RMSF of **11** and **12** with c-Raf.

From Figure 18, it is clear that ligand **11** fluctuates less in the region of two chlorine atoms, and **12** has a strong deviation of one chlorine atom by 4.5 Å (Figure 18). This is due to the fact that, in the 3,5-dichloroadamantyl fragment, one of the chlorine atoms is stabilized by a halogen connection with Lys431, while in **12** the presence of a spacer makes the molecule more flexible and one chlorine atom cannot constantly interact with this amino acid residue, while Lys431 interacts with the carbonyl urea group through a water molecule.

**Figure 19:**
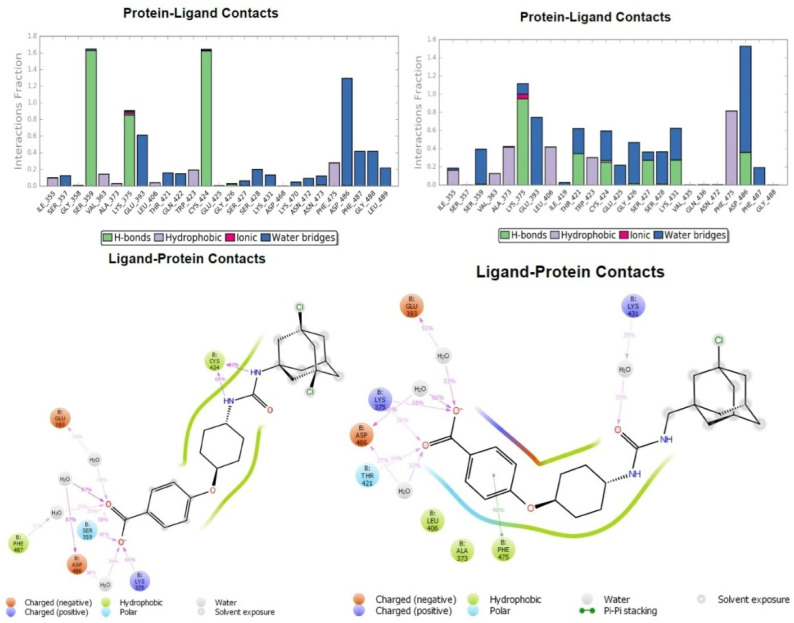
Protein–ligand contacts of **11** and **12** with c-Raf.

The urea group of ligand **11** forms stable hydrogen bonds with Cys424 and Ser359, which persist almost throughout the entire simulation time, while the ureide fragment **12** does not interact with this amino acid residue at all (Figure 19). Due to the bridge between adamantane and the urea group, the inhibitor molecule is able to form pi-stacking with Phe475.

Compound **11** forms a more stable complex than compound **12**, even though the latter has a spacer between the adamantyl moiety and the urea group. This is probably due to additional interactions of the second chlorine atom in adamantane, which forms a fairly strong (2.58 A) halogen bond with Lys431 residue. It cannot be said with certainty that this inhibitor is active against c-Raf, but data from molecular docking and molecular dynamics suggest that compound 11 is a potential inhibitor of c-Raf.

## 4. Materials and Methods

### 4.1. Chemistry

Triethylamine (BioUltra ≥99.5%, CAS 121-44-8), diphenylphosphoryl azide (97%, CAS 26386-88-9), 1,8-diazabicyclo[5.4.0]undec-7-ene (98%, CAS 6674-22-2), 1,2-diaminoethane (≥99%, CAS 107-15-3), 1,3-diaminopropane (≥99%, CAS 109-76-2), 1,4-diaminobutane (99%, CAS 110-60-1), 1,5-diaminopentane (≥97%, CAS 462-94-2), 1,6-diaminohexane (98%, CAS 124-09-4), 1,7-diaminoheptane (98%, CAS 646-19-5), 1,8-diaminooctane (98%, CAS 373-44-4), 1,9-diaminononane (97%, CAS 646-24-2), 1,10-diaminooctane (97%, CAS 646-25-3), and 3-hydroxyadamantane-1-carboxylic acid (97%, CAS 42711-75-1) manufactured by Sigma-Aldrich (St. Louis, MO, USA) were used without purifying.

Diethyl ether, toluene, benzene, and thionyl chloride were purified by well-known methods. Trans-4-amino(cyclohexyloxy)benzoic acid **10** was obtained according to the method described in work [104].

### 4.2. Equipment

Purification of the obtained adamantile-containing derivatives **2**, **3**, **4** and **5** was performed on a Pure C-815 Flash Advanced chromatographic system (Buchi Labortechnik AG, Flawil, Switzerland).

The structure of the obtained compounds was confirmed by GC-MS (Figures S1–S8 in the Supplementary Materials), ^1^H and ^13^C NMR spectroscopy (Figures S9–S30 in the Supplementary Materials) and elemental analysis. Mass spectra were recorded on an Agilent GC 7820A/MSD 5975 chromatography-mass spectrometer (Agilent Technologies, Santa Clara, CA, USA) in fullscan (EI) mode. NMR ^1^H and ^13^C was performed on Bruker DPX 300 (Bruker Corporation, Billerica, MA, USA) in a DMSO-d_6_ solvent; chemical shifts ^1^H were given relative to SiMe_4_. Elemental analysis was performed on a Perkin-Elmer Series II 2400 instrument (Perkin-Elmer, Waltham, MA, USA). Melting points were determined using an OptiMelt MPA100 instrument (Stanford Research Systems, Sunnyvale, CA, USA).

### 4.3. Molecular Docking

#### 4.3.1. sEH (C-Terminal Domain)

Molecular docking of the compounds was carried out in a 3D model of the sEH – t-AUCB complex (PDB: 5AM3) (substances used for X-ray structural analysis of proteins and the t-AUCB molecule were previously removed from the model). Compounds and protein were pre-prepared using the Maestro Schrödinger 11.1 program. The docking procedure was carried out in the same program using the Extra Precision protocol (coordinates of the center: x = 14.9 Å, y = 10.57 Å, z = 15.59 Å, completeness: = 20, energy range: = 4). Ligand–protein complexes with the best scoring functions were selected.

#### 4.3.2. p38 MAPK (Allosteric Site)

Molecular docking of the compounds was carried out in a 3D model of the p38 – inhibitor complex (PDB: 1KV1) (substances used for X-ray structural analysis of proteins and the inhibitor molecule were previously removed from the model). Compounds and protein were pre-prepared using the Maestro Schrödinger 11.1 program. The docking procedure was carried out in the same program using the Extra Precision protocol (coordinates of the center: x = 28.84 Å, y = 29.11 Å, z = 11.32 Å, completeness: = 20, energy range: = 4). Ligand–protein complexes with the best scoring functions were selected.

#### 4.3.3. p38 MAPK (Active Site)

Molecular docking of the compounds was carried out in a 3D model of the p38 – Sorafenib complex (PDB: 3HEG) (substances used for X-ray structural analysis of proteins and the sorafenib molecule were previously removed from the model). Compounds and protein were pre-prepared using the Maestro Schrödinger 11.1 program. The docking procedure was carried out in the same program using the Extra Precision protocol (coordinates of the center: x = 1.26 Å, y = 1.38 Å, z = 21.37 Å, completeness: = 20, energy range: = 4). Ligand–protein complexes with the best scoring functions were selected.

#### 4.3.4. c-Raf

Molecular docking of the compounds was carried out in a 3D model of the c-Raf – inhibitor complex (PDB: 3OMV) (substances used for X-ray structural analysis of proteins and the inhibitor molecule were previously removed from the model). Compounds and protein were pre-prepared using the Maestro Schrödinger 11.1 program. The docking procedure was carried out in the same program using the Extra Precision protocol (coordinates of the center: x = 31.17 Å, y = 35.45 Å, z = 41.31 Å, completeness: = 20, energy range: = 4). Ligand–protein complexes with the best scoring functions were selected.

### 4.4. MM/GBSA Calculations

Compounds that were successfully docked were subjected to a rescoring process using the MM-GBSA method implemented in Prime (Schrödinger, LLC, New York, NY, 2018).

The binding free energy of MM/GBSA was estimated by minimizing the protein–ligand complex to eliminate errors that occur when using a rigid protein structure in docking procedures. Protein flexibility was allowed for residues up to 5 Å from the ligand. Hydrogen interactions were considered acceptable when the distance between atoms was less than 3 Å.

### 4.5. Molecular Dynamics

Simple MD simulations were performed using Desmond 4.1 [105] with the OPLS3 force field [106]. The complexes were solvated in orthorhombic boxes using the TIP3P aqueous model. Ions were added to neutralize the charges. The systems were minimized and equilibrated at a temperature of 300 K and a pressure of 1.013 bar. The system was modeled as an NPT ensemble; a Nose–Hover thermostat and a Martin–Tobi–Klein barostat were used. The integration step was chosen to be 2 fs. To maintain the rigidity of hydrogen-heavy atom bonds, the SHAKE algorithm was used. A cutoff radius of 9 Å was set for short-range Coulomb interactions, and a smooth Ewald particle mesh was used for long-range interactions. For each system, we performed MD for 30 ns, with a detection range of 1.2 ps for energy and 30 ps for trajectory frames. Visualization and analysis of MD trajectories were performed using Desmond modeling tools in Maestro.

### 4.6. Procedure for Synthesis Series of 1,3-Disubstituted Ureas ***7a***–***7i*** and ***9***, ***11***

3,5-Dihydroxyadamantan-1-yl carboxylic acid (2)

To a solution of 5 g (89 mmol) KOH in 350 mL of water, 12.8 g (81 mmol) of KMnO_4_ was added. After heating the resulting solution to 50 °C, 15.9 g (81 mmol) of 3-hydroxyadamantan-1-yl carboxylic acid **1** was added in portions. Then, the reaction mixture was heated to a boil and kept at this temperature for 5 h. After cooling, 6N HCl was added to pH 8–9, and then sodium hydrosulfite was also added. The precipitated unreacted acid **1** was filtered off, and the filtrate was saturated with sodium chloride. The product was extracted twice with a mixture of solvents (ethyl acetate 3:1 methanol), followed by separation and concentration of the organic layer. The yield of the crude product was 47%. The resulting mixture of acids was dissolved in water. The admixture of monohydroxy acid was filtered off, and the saturated aqueous solution was concentrated to obtain the target product with a purity of 99%. The yield was 6.36 g (30.0 mmol, 37%). Unreacted acid **1** was re-introduced into the oxidation reaction to obtain another 7.75 g (36.5 mmol, 45%) of acid 2. Mass spectrum, *m*/*z* (Irel. %): 212 (18% [M]^+^), 167 (75% [(HO)_2_-Ad]^+^). Calc. for C_11_H_16_O_4_: C 62.25; H 7.60. Found: C 62.28; H 7.64. M = 212.25.

3,5-Dichloroadamantan-1-yl Carboxylic Acid (4)

To a solution of 10 g (47 mmol) of 3,5-dihydroxyadamantan-1-yl carboxylic acid **2** and 40 mL of dried benzene, 18.5 g (155 mmol) of thionyl chloride was added and stirred at room temperature for 24 h. Thionyl chloride was distilled off with benzene, followed by concentration of the anhydride (**3**) in vacuo. The obtained acid chloride without isolation and purification was suspended in a 1M NaOH aqueous solution (100 mL) and stirred for 8 h at room temperature. The resulting solution was washed with dichloromethane (100 mL) and acidified with hydrochloric acid to pH 1.0. The precipitated solid was collected by filtration, washed with water (200 mL) and dried. Acid **4** was recrystallized from the solvent—ethyl acetate/hexane. The yield was 7.96 g (68%), white crystals, m.p. 162–163 °C. Mass spectrum was *m*/*z* (Irel. %): 266 (1% [M]^+^), 231 (3% [(Cl)_2_-Ad-C(O)]^+^), 205 (100% [(Cl)_2_-Ad]^+^). Calc. for C_11_H_13_Cl_2_O: C 49.38; H 4.90. Found: C 49.40; H 4.92. M = 267.57.

3,5-Dichloroadamantan-1-yl Isocyanate (5)

To a mixture of 7.0 g (28 mmol) of 3,5-dichloroadamantan-1-yl carboxylic acid **4** and 2.8 g (28 mmol) of triethylamine in 70 mL of anhydrous toluene, 7.7 g (28 mmol) of diphenylphosphoryl azide was added dropwise over 30 min at room temperature. Then, the reaction mixture was heated to a boil and kept for 30 min until the complete cessation of nitrogen evolution. Toluene was evaporated, the product was taken from the reaction mixture with anhydrous diethyl ether. The yield was 6.1 g (88%), white crystals, m.p. 105 °C. Mass spectrum was *m*/*z* (Irel. %): 245 (70% [M]^+^), 210 (100% [Cl-Ad-NCO]^+^), 175 (28% [Ad-NCO]^+^). Calc. for C_11_H_13_Cl_2_NO_2_: C 53.68; H 5.32. Found: C 53.70; H 5.36. M = 246.13.

1,1’-(Ethane-1,2-diyl)bis(3-(3,5-dichloroadamantan-1-yl)urea)

^1^H NMR (400 MHz, DMSO-*d*_6_, δ) ppm: 1.67–1.75 m (4H, Ad), 1.90 d (4H, Ad, *J* = 12.0 Hz), 1.94 q (8H, Ad, *J* = 12.0 Hz), 2.26–2.42 m (10H, Ad), 2.88–2.97 m (4H, NH-CH_2_-CH_2_-NH), 5.72 s (2H, NH-(CH_2_)_2_-NH), 6.01 s (2H, NH-Ad). ^13^C NMR (100 MHz, DMSO-*d*_6_, δ) ppm: 32.33 (C^3^, C^17^), 38.65 (C^2^, C^12^, C^13^, C^16^), 44.46 (C^4^, C^10^, C^18^, C^24^), 50.01 (C^8^, C^9^, C^22^, C^23^), 54.37 (C^6^, C^20^), 55.34 (C^1^, C^15^), 67.28 (C^5^, C^7^, C^19^, C^21^), 157.47 (C^11^, C^14^). Calc. for C_24_H_34_Cl_4_N_4_O_2_: C 52.19; H 6.20; N 10.14. Found: C 52.26; H 6.25; N 10.18. M = 552.36.

1,1’-(Propan-1,3-diyl)bis(3-(3,5-dichloroadamantan-1-yl)urea)

^1^H NMR (400 MHz, DMSO-*d*_6_, δ) ppm: 1.37 p (2H, NH-CH_2_-CH_2_-CH_2_-NH, *J* = 6.6 Hz), 1.67–1.74 m (4H, Ad), 1.94 q (8H, Ad, *J* = 11.9 Hz), 2.12 d (4H, Ad, *J* = 11.5 Hz), 2.41–2.25 m (10H, Ad), 2.85–3.96 m (4H, NH-CH_2_-CH_2_-CH_2_-NH), 5.75 s (2H, NH-(CH_2_)_3_-NH), 6.01 s (2H, NH-Ad). ^13^C NMR (100 MHz, DMSO-*d*_6_, δ) ppm: 31.46 (C^13^), 32.33 (C^3^, C^18^), 36.73 (C^12^, C^14^), 38.63 (C^2^, C^17^), 44.46 (C^4^, C^10^, C^19^, C^25^), 50.01 (C^8^, C^9^, C^23^, C^24^), 54.35 (C^6^, C^21^), 55.35 (C^1^, C^16^), 67.31 (C^5^, C^7^, C^20^, C^22^), 157.46 (C^11^, C^15^). Calc. for C_25_H_36_Cl_4_N_4_O_2_: C 53.02; H 6.41; N 9.89. Found: C 53.05; H 6.44; N 9.93. M = 566.39.

1,1’-(Butan-1,4-diyl)bis(3-(3,5-dichloroadamantan-1-yl)urea)

^1^H NMR (400 MHz, CDCl_3_, δ) ppm: 1.25 s (4H, NH-CH_2_-CH_2_-CH_2_-CH_2_-NH), 1.58 s (4H, Ad), 1.88 s (4H, Ad), 1.99 s (6H, Ad), 2.26 d (4H, Ad, *J* = 11.5 Hz), 2.39–2.43 m (8H, Ad), 2.89 s (2H, NH-CH_2_-CH_2_-CH_2_-CH_2_-NH), 2.97 s (2H, NH-CH_2_-CH_2_-CH_2_-CH_2_-NH), 6.24 s (2H, NH-(CH_2_)_4_-NH), 7.26 s (2H, NH-Ad). ^13^C NMR (100 MHz, CDCl_3_, δ) ppm: 26.53 (C^13^, C^14^), 29.68 (C^3^, C^19^), 38.81 (C^12^, C^15^), 39.89 (C^2^, C^18^), 44.42 (C^4^, C^10^, C^20^, C^26^), 49.73 (C^8^, C^9^, C^24^, C^25^), 55.16 (C^6^, C^22^), 55.72 (C^1^, C^17^), 64.53 (C^5^, C^7^, C^21^, C^23^), 159.23 (C^11^, C^16^). Calc. for C_26_H_38_Cl_4_N_4_O_2_: C 53.80; H 6.60; N 9.65. Found: C 53.84; H 6.65; N 9.68. M = 580.42.

1,1’-(Pentan-1,5-diyl)bis(3-(3,5-dichloroadamantan-1-yl)urea)

^1^H NMR (400 MHz, CDCl_3_, δ) ppm: 1.31 s (2H, NH-CH_2_-CH_2_-CH_2_-CH_2_-CH_2_-NH), 1.41–1.50 m (4H, NH-CH_2_-CH_2_-CH_2_-CH_2_-CH_2_-NH), 1.82 s (4H, Ad), 1.97 s (8H, Ad), 2.22 d (4H, Ad, *J* = 11.9 Hz), 2.34–2.46 m (10H, Ad), 2.88 s (2H, NH-CH_2_-CH_2_-CH_2_-CH_2_-CH_2_-NH), 2.96 s (2H, NH-CH_2_-CH_2_-CH_2_-CH_2_-CH_2_-NH), 5.84 s (2H, NH-(CH_2_)_5_-NH), 7.26 s (2H, NH-Ad). ^13^C NMR (100 MHz, CDCl_3_, δ) ppm: 26.03 (C^14^), 29.68 (C^13^, C^15^), 32.17 (C^3^, C^20^), 39.17 (C^2^, C^19^), 39.50 (C^12^, C^16^), 44.68 (C^4^, C^10^, C^21^, C^27^), 50.18 (C^8^, C^9^, C^25^, C^26^), 54.60 (C^6^, C^23^), 55.41 (C^1^, C^18^), 65.17 (C^5^, C^7^, C^22^, C^24^), 158.29 (C^11^, C^17^). Calc. for C_27_H_40_Cl_4_N_4_O_2_: C 54.55; H 6.78; N 9.43. Found: C 54.58; H 6.80; N 9.46. M = 594.44.

1,1’-(Hexane-1,6-diyl)bis(3-(3,5-dichloroadamantan-1-yl)urea)

^1^H NMR (400 MHz, DMSO-*d*_6_, δ) ppm: 1.11–1.24 m (2H, NH-CH_2_-CH_2_-CH_2_-CH_2_-CH_2_-CH_2_-NH), 1.27–1.34 m (2H, NH-CH_2_-CH_2_-(CH_2_)_2_-CH_2_-CH_2_-NH), 1.71 s (4H, Ad), 1.94 q (8H, Ad, *J* = 12.1 Hz), 2.11 d (4H, Ad, *J* = 11.7 Hz), 2.28–2.40 m (10H, Ad), 2.88–2.96 m (4H, NH-CH_2_-(CH_2_)_4_-CH_2_-NH), 5.69 s (2H, NH-(CH_2_)_6_-NH), 5.85 s (2H, NH-Ad). ^13^C NMR (100 MHz, DMSO-*d*_6_, δ) ppm: 24.30 (C^14^, C^15^), 30.12 (C^13^, C^16^), 32.33 (C^3^, C^21^), 38.65 (C^2^, C^20^), 39.24 (C^12^, C^17^), 44.45 (C^4^, C^10^, C^22^, C^28^), 50.03 (C^8^, C^9^, C^26^, C^27^), 55.33 (C^6^, C^24^), 55.41 (C^1^, C^19^), 67.31 (C^5^, C^7^, C^23^, C^25^), 157.35 (C^11^, C^18^). Calc. for C_28_H_42_Cl_4_N_4_O_2_: C 55.27; H 6.96; N 9.21. Found: C 55.30; H 6.98; N 9.25. M = 608.47.

1,1’-(Heptan-1,7-diyl)bis(3-(3,5-dichloroadamantan-1-yl)urea)

^1^H NMR (400 MHz, DMSO-*d*_6_, δ) ppm: 1.17–1.25 m (6H, NH-CH_2_-CH_2_-CH_2_-CH_2_-CH_2_-CH_2_CH_2_-NH), 1.27–1.34 m (4H, NH-CH_2_-CH_2_-(CH_2_)_3_-CH_2_-CH_2_-NH), 1.71 d (4H, Ad, *J* = 3.2 Hz), 1.93 q (8H, Ad, *J* = 12.0 Hz), 2.11 d (4H, Ad, *J* = 11.9 Hz), 2.27–2.40 m (10H, Ad), 2.90 q (4H, NH-CH_2_-(CH_2_)_5_-CH_2_-NH, *J* = 6.5 Hz), 5.69 t (2H, NH-(CH_2_)_7_-NH, *J* = 5.7 Hz), 5.86 s (2H, NH-Ad). ^13^C NMR (100 MHz, DMSO-*d*_6_, δ) ppm: 26.86 (C^14^, C^16^), 30.33 (C^13^, C^17^), 31.66 (C^15^), 32.33 (C^3^, C^22^), 38.66 (C^2^, C^21^), 39.23 (C^12^, C^18^), 44.46 (C^4^, C^10^, C^23^, C^29^), 46.11 (C^6^, C^25^), 50.04 (C^8^, C^9^, C^27^, C^28^), 55.34 (C^1^, C^20^), 67.30 (C^5^, C^7^, C^24^, C^26^), 157.36 (C^11^, C^19^). Calc. for C_29_H_44_Cl_4_N_4_O_2_: C 55.96; H 7.12; N 9.00. Found: C 55.98; H 7.16; N 9.04. M = 622.50.

1,1’-(Octane-1,8-diyl)bis(3-(3,5-dichloroadamantan-1-yl)urea)

^1^H NMR (400 MHz, DMSO-*d*_6_, δ) ppm: 1.16–1.26 m (8H, NH-CH_2_-CH_2_-CH_2_-CH_2_-CH_2_-CH_2_-CH_2_CH_2_-NH), 1.27–1.36 m (4H, NH-CH_2_-CH_2_-(CH_2_)_4_-CH_2_-CH_2_-NH), 1.71 d (4H, Ad, *J* = 3.1 Hz), 1.94 q (8H, Ad, *J* = 12.1 Hz), 2.11 d (4H, Ad, *J* = 11.6 Hz), 2.26–2.41 m (10H, Ad), 2.90 q (4H, NH-CH_2_-(CH_2_)_6_-CH_2_-NH, *J* = 6.4 Hz), 5.68 t (2H, NH-(CH_2_)_8_-NH, *J* = 5.7 Hz), 5.85 s (2H, NH-Ad). ^13^C NMR (100 MHz, DMSO-*d*_6_, δ) ppm: 26.80 (C^14^, C^17^), 29.22 (C^15^, C^16^), 30.37 (C^13^, C^18^), 32.33 (C^3^, C^23^), 38.66 (C^2^, C^22^), 39.23 (C^12^, C^19^), 44.46 (C^4^, C^10^, C^24^, C^30^), 50.04 (C^8^, C^9^, C^28^, C^29^), 53.42 (C^6^, C^26^), 55.34 (C^1^, C^21^), 67.30 (C^5^, C^7^, C^25^, C^27^), 157.36 (C^11^, C^20^). Calc. for C_30_H_46_Cl_4_N_4_O_2_: C 56.61; H 7.28; N 8.80. Found: C 56.64; H 7.30; N 8.84. M = 636.52.

1,1’-(Nonan-1,9-diyl)bis(3-(3,5-dichloroadamantan-1-yl)urea)

^1^H NMR (400 MHz, DMSO-*d*_6_, δ) ppm: 1.12.25 m (10H, NH-CH_2_-CH_2_-CH_2_-CH_2_-CH_2_-CH_2_-CH_2_-CH_2_-CH_2_-NH), 1.25–1.35 m (4H, NH-CH_2_-CH_2_-(CH_2_)_5_-CH_2_-CH_2_-NH), 1.70 d (4H, Ad, *J* = 3.2 Hz), 1.93 q (8H, Ad, *J* = 11.9 Hz), 2.10 d (4H, Ad, *J* = 11.6 Hz), 2.25–2.42 m (10H, Ad), 2.90 q (4H, NH-CH_2_-(CH_2_)_7_-CH_2_-NH, *J* = 6.4 Hz), 5.68 t (2H, NH-(CH_2_)_9_-NH, *J* = 5.7 Hz), 5.86 s (2H, NH-Ad). ^13^C NMR (100 MHz, DMSO-*d*_6_, δ) ppm: 26.83 (C^14^, C^18^), 29.15 (C^15^, C^17^), 29.49 (C^16^), 30.39 (C^13^, C^19^), 32.33 (C^3^, C^24^), 38.66 (C^2^, C^23^), 39.22 (C^12^, C^20^), 44.46 (C^4^, C^10^, C^25^, C^31^), 50.04 (C^8^, C^9^, C^29^, C^30^), 51.17 (C^6^, C^27^), 55.34 (C^1^, C^22^), 67.29 (C^5^, C^7^, C^26^, C^28^), 157.37 (C^11^, C^21^). Calc. for C_31_H_48_Cl_4_N_4_O_2_: C 57.23; H 7.44; N 8.61. Found: C 57.26; H 7.48; N 8.65. M = 650.55.

1,1’-(Decan-1,10-diyl)bis(3-(3,5-dichloroadamantan-1-yl)urea)

^1^H NMR (400 MHz, DMSO-*d*_6_, δ) ppm: 1.10–1.25 m (12H, NH-CH_2_-CH_2_-CH_2_-CH_2_-CH_2_-CH_2_-CH_2_-CH_2_-CH_2_-CH_2_-NH), 1.26–1.34 m (4H, NH-CH_2_-CH_2_-(CH_2_)_6_-CH_2_-CH_2_-NH), 1.70 d (4H, Ad, *J* = 3.2 Hz), 1.93 q (8H, Ad, *J* = 11.9 Hz), 2.10 d (4H, Ad, *J* = 11.5 Hz), 2.26–2.41 m (10H, Ad), 2.90 q (4H, NH-CH_2_-(CH_2_)_8_-CH_2_-NH, *J* = 6.4 Hz), 5.68 t (2H, NH-(CH_2_)_8_-NH, *J* = 5.7 Hz), 5.85 s (2H, NH-Ad). ^13^C NMR (100 MHz, DMSO-*d*_6_, δ) ppm: 26.85 (C^14^, C^19^), 29.22 (C^15^, C^18^), 29.45 (C^13^, C^20^), 30.39 (C^16^, C^17^), 32.33 (C^3^, C^25^), 38.67 (C^2^, C^24^), 39.22 (C^12^, C^21^), 44.46 (C^4^, C^10^, C^26^, C^32^), 46.11 (C^6^, C^28^), 50.04 (C^8^, C^9^, C^30^, C^31^), 55.34 (C^1^, C^23^), 67.29 (C^5^, C^7^, C^27^, C^29^), 157.36 (C^11^, C^22^). Calc. for C_32_H_50_Cl_4_N_4_O_2_: C 57.83; H 7.58; N 8.43. Found: C 57.86; H 7.60; N 8.46. M = 664.58.

1,3-Bis-(3,5-dichloroadamantan-1-yl)urea

^1^H NMR (400 MHz, DMSO-*d*_6_, δ) ppm: 1.69 d (4H, Ad, *J* = 3.2 Hz), 1.93 q (8H, Ad, *J* = 11.8 Hz), 2.09 d (4H, Ad, *J* = 11.1 Hz), 2.23–2.45 m (10H, Ad), 5.90 s (2H, 2NH). ^13^C NMR (100 MHz, DMSO-*d*_6_, δ) ppm: 32.30 (C^3^, C^14^), 38.53 (C^2^, C^13^), 44.42 (C^4^, C^10^, C^15^, C^21^), 49.82 (C^8^, C^9^, C^19^, C^20^), 54.34 (C^6^, C^17^), 55.29 (C^1^, C^12^), 67.23 (C^5^, C^7^, C^16^, C^18^), 153.97 (C^11^). Calc. for C_21_H_28_Cl_2_N_2_O: C 54.10; H 6.05; N 6.01. Found: C 54.14; H 6.08; N 6.05. M = 466.27.

4-((4-(3-(3,5-Dichloroadamantan-1-yl)ureido)cyclohexyl)oxy)benzoic Acid

^1^H NMR (400 MHz, DMSO-*d_6_*, δ) ppm: 0.92–1.49 m (4H, CH_2_ cyclohex), 1.70 d (2H, Ad, *J* = 6.2 Hz), 1.76–1.87 m (2H, CH_2_ cyclohex), 1.93 q (4H, Ad, *J* = 11.9 Hz), 2.09 t (2H, Ad, *J* = 9.9 Hz), 2.16–2.29 m (2H, CH_2_ cyclohex), 2.30–2.38 m (5H, Ad), 3.07 qd (1H, CH-NH, *J* = 39.0, 7.1 Hz), 4.34–4.45 m (1H, CH-O-), 5.74 d (1H, NH-CH, *J* = 7.7 Hz), 5.88 s (1H, NH-Ad), 6.90–7.42 m (4H, arom),12.56 s (1H, COOH). ^13^C NMR (100 MHz, DMSO-*d*_6_, δ) ppm: 30.11 (C^13^, C^17^), 30.64(C^14^, C^16^), 32.33 (C^3^), 38.52 (C^2^), 44.44 (C^4^, C^10^), 44.41 (C^12^), 49.82 (C^8^, C^9^), 49.89 (C^6^), 55.28 (C^1^), 67.23 (C^5^, C^7^), 74.78 (C^15^), 115.53 (C^19^, C^23^), 125.19 (C^21^), 131.81 (C^20^, C^22^), 156.76 (C^11^), 161.54 (C^18^), 167.41 (C^24^). Calc. for C_24_H_30_Cl_2_N_2_O_4_: C 59.88; H 6.28; N 5.82. Found: C 59.90; H 6.30; N 5.84. M = 481.41.

## 5. Conclusions

Three targets were selected for which the urea class may be active. The signaling pathways in which these enzymes are involved were considered. 1,3-Dichloroadamantyl-containing ureas and diureas were synthesized, which can be potential inhibitors of p38 MAPK, c-Raf, and sEH. The ability of the synthesized compounds to form stable protein-ligand complexes with high specificity was shown by molecular docking and calculation of the free energies of MM/GBSA binding. The two chlorine atoms in the adamantyl moiety promoted the formation of additional Cl-π interactions, which can have a positive effect on biochemical activity. The synthesized compound 11, which was chosen as the main one for molecular docking, had a lower melting point than its analogue with one chlorine atom in the adamantyl fragment or two methyl substituents, which may also contribute to improved metabolic characteristics in the future.

Molecular dynamics simulations showed significant advantages in the presence of a spacer between the adamantyl moiety and the urea group, which provided additional conformational mobility in the active site of the enzymes. It cannot be said with certainty that the synthesized compounds are successful in inhibiting all three targets; however, the data obtained are useful in the development of more effective inhibitors. It is worth thinking about the synthesis and study of molecules containing two or more chlorine atoms in the adamantyl radical, as well as the bridge between this fragment and the urea group.

In the future, work will be continued with the study of inhibitory activity in vitro.

## Figures and Tables

**Table 1:** Lipophilicity coefficients, melting points and yields of synthesized compounds **7a**–**i**, **9**, **11**.

No.	Mr	cLog*P* *	*t*_m.p._, °C	Yield, %
**7a**	552	5.47	202	60
**7b**	566	5.74	145	55
**7c**	580	6.01	211	71
**7d**	594	6.52	73	56
**7e**	608	7.03	86	51
**7f**	622	7.53	132	78
**7g**	636	8.04	182	69
**7h**	650	8.43	163	90
**7i**	664	8.72	126	76
**9**	466	6.04	230	49
**11**	481	4.81	209	49

* Calculated using software Molinspiration (http://www.molinspiration.com, accessed on 10 October 2022) © Molinspiration Cheminformatics.

## Data Availability

Data is contained within the article or Supplementary Materials.

## Supplementary Materials

The following supporting information can be downloaded at: https://www.mdpi.com/article/10.3390/ijms25010338/s1.
