# Supplementary material for: 1,3-Dichloroadamantyl-Containing Ureas as Potential Triple Inhibitors of Soluble Epoxide Hydrolase, p38 MAPK and c-Raf
Source: Int J Mol Sci. 2023 Dec 26;25(1):338. doi: 10.3390/ijms25010338 (PMC10779153; doi:10.3390/ijms25010338)

1,3-Dichloroadamantyl-Containing Ureas as Potential Triple Inhibitors of Soluble Epoxide Hydrolase, p38 MAPK and c-Raf

Boris P. Gladkikh[1], Dmitriy V. Danilov[1], Vladimir S. D’yachenko[1,2,*], Gennady M. Butov[1,2]

1 Department of Technology of Organic and Petrochemical Synthesis, Volgograd State Technical University,

Volgograd 400005, Russia; gladkih-boris@mail.ru (B.P.G.); danilov.dmitry.vlz@yandex.ru (D.V.D.);

gmbutov@mail.ru (G.M.B.)

2 Department of Chemistry, Technology and Equipment of Chemical Industry, Volzhsky Polytechnic

Institute (Branch), Volgograd State Technical University (VSTU), Volzhsky 404121, Russia

* Correspondence: v.s.dyachenko@vstu.ru

Supplementary materials

^{1}H NMR, ^{13}C NMR and Mass spectra

Figure S1. Chromatogram of compound **2**

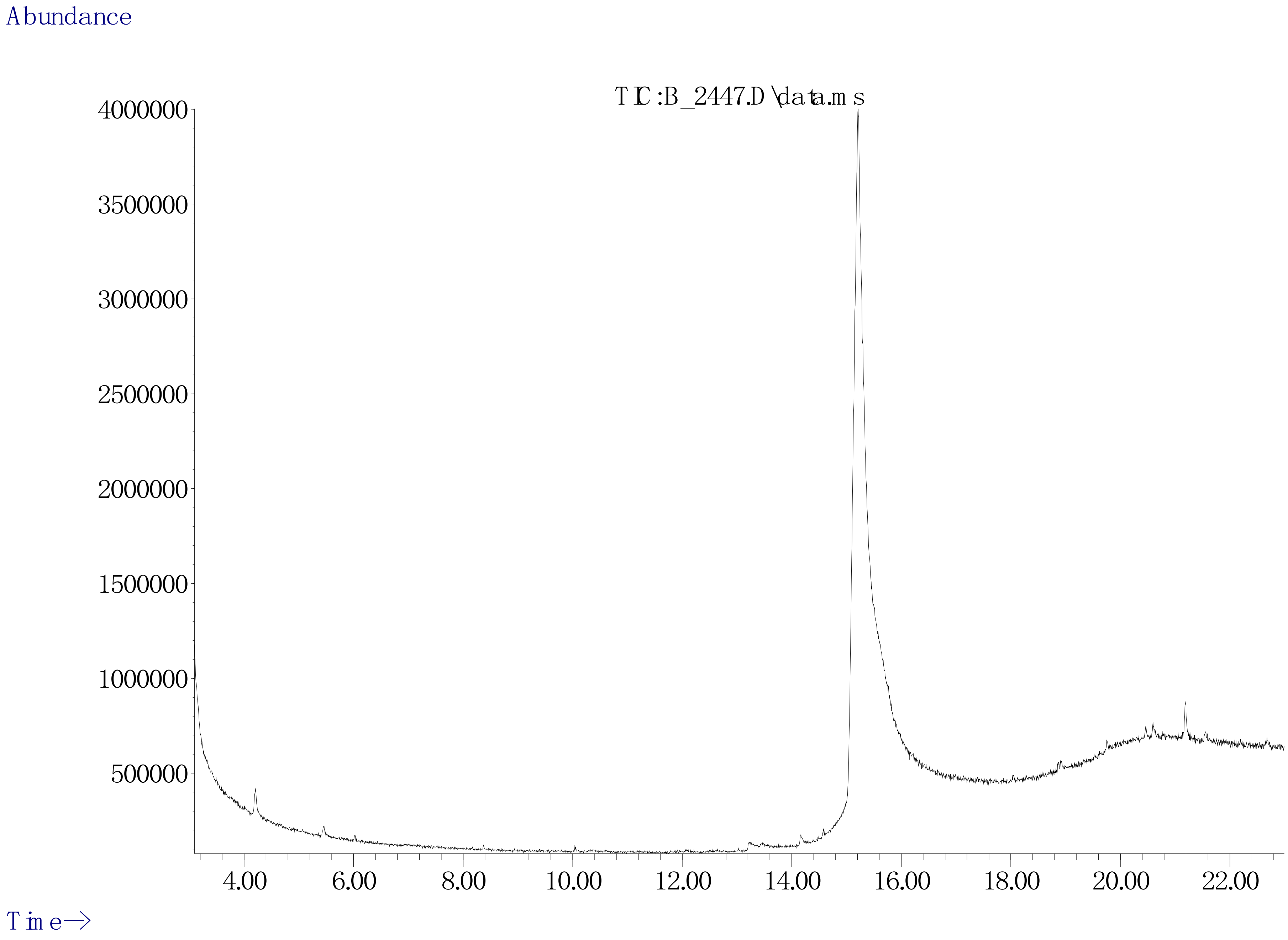

Figure S2. Mass spectrum of compound **2**

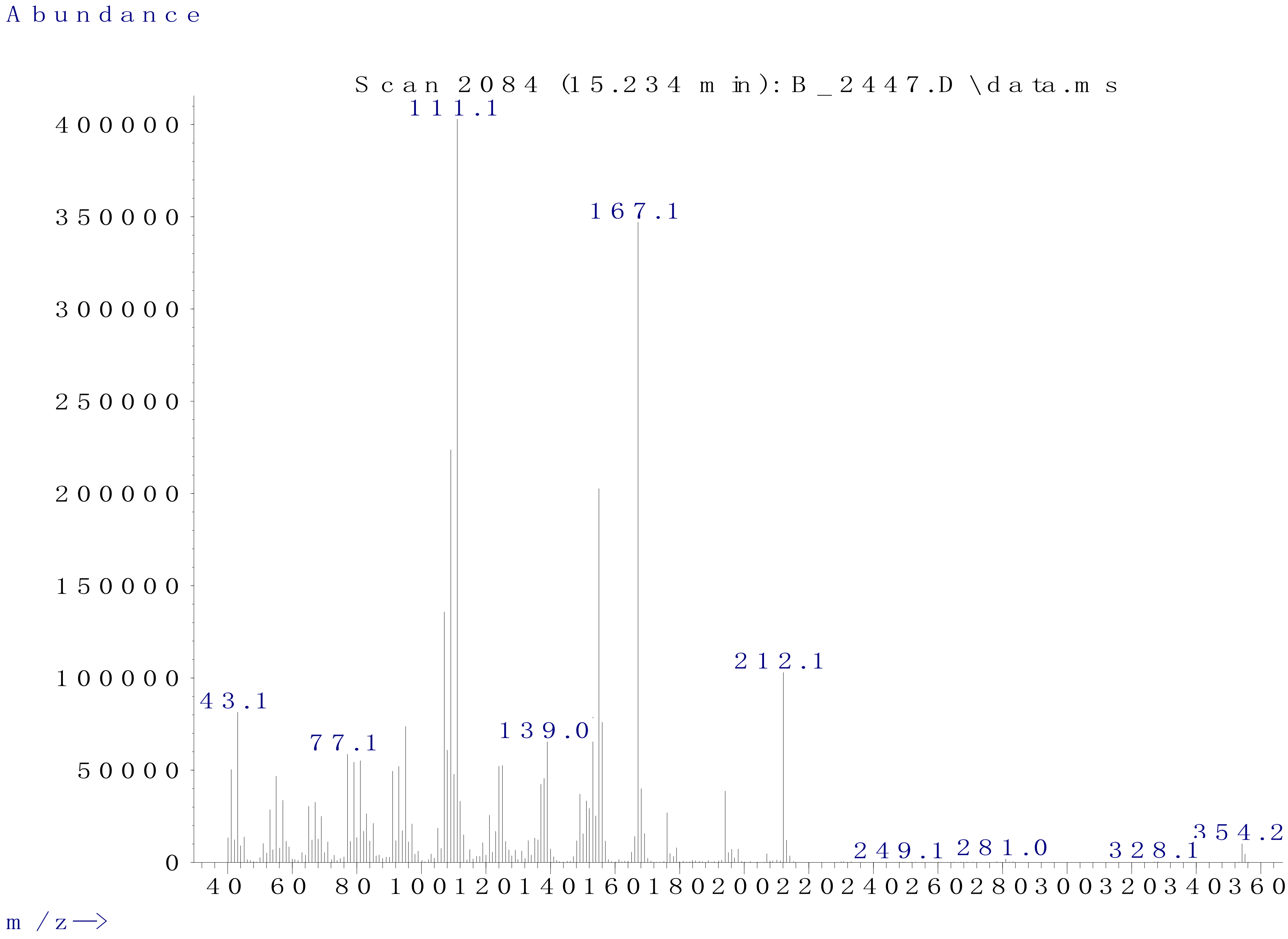

Figure S3. Chromatogram of compound **3**

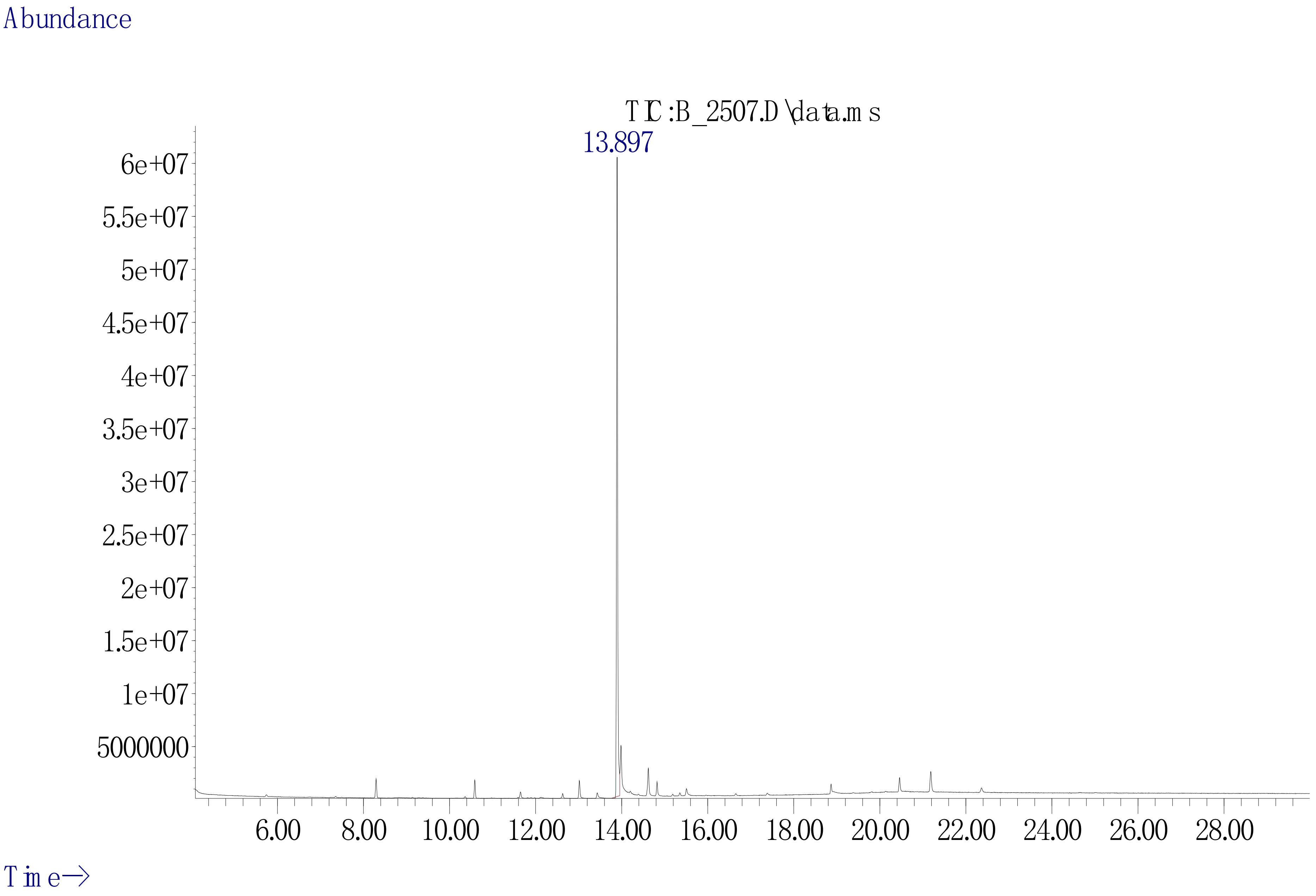

Figure S4. Mass spectrum of compound **3**

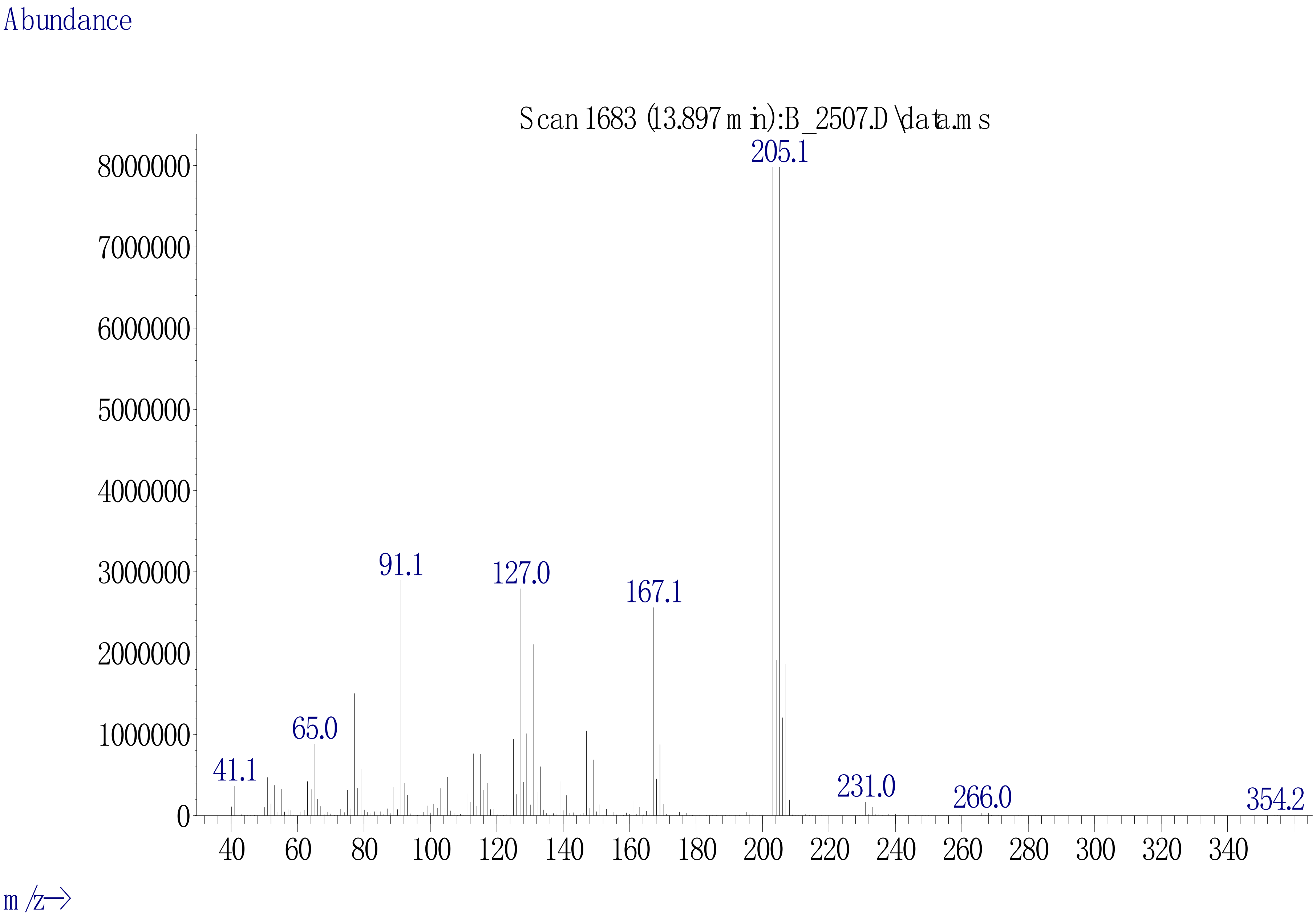

Figure S5. Chromatogram of compound **4**

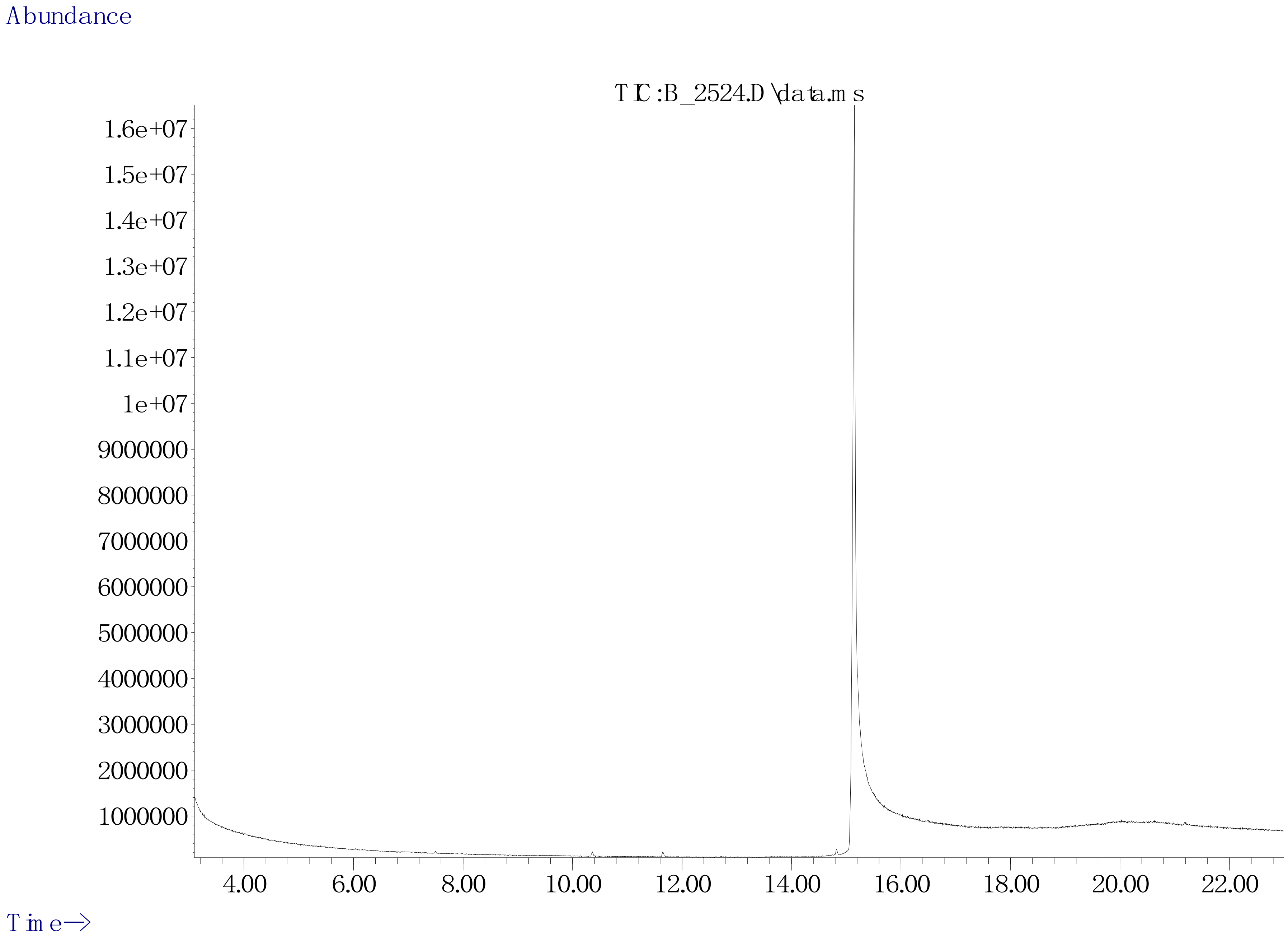

Figure S6. Mass spectrum of compound **4**

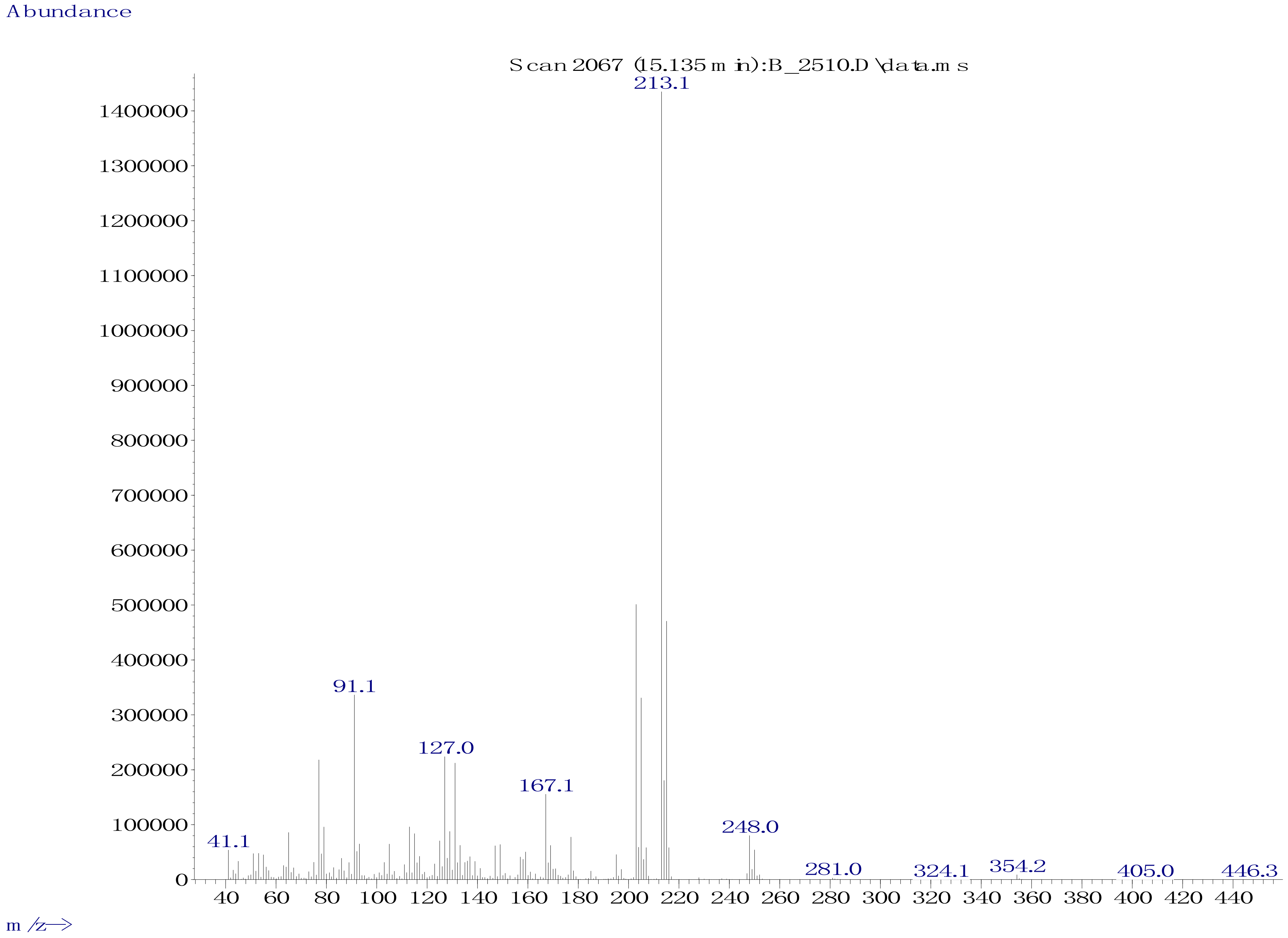

Figure S7. Chromatogram of compound **5**

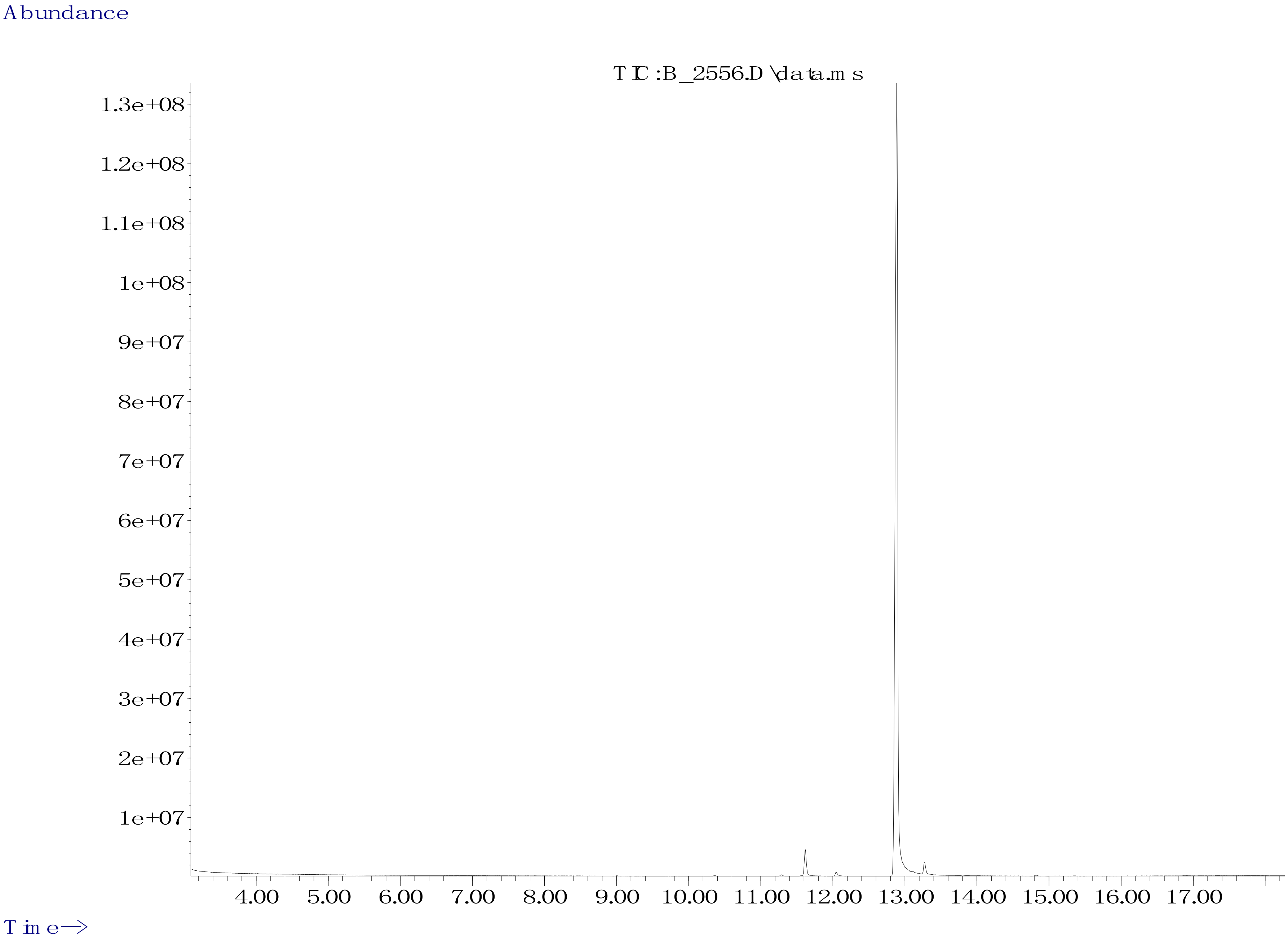

Figure S8. Mass spectrum of compound **5**

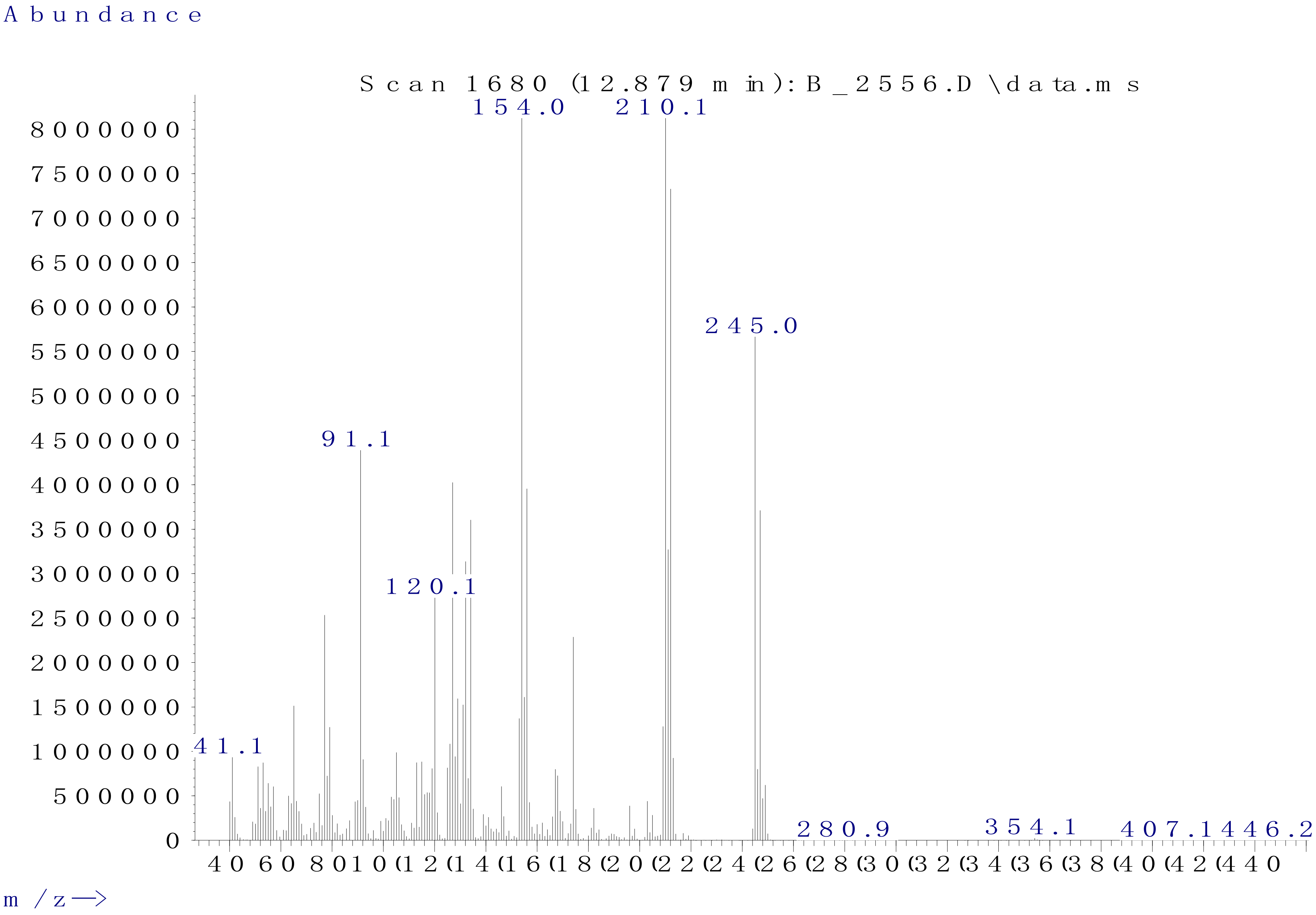

Figure S9. ^{1}H NMR of compound **7a**

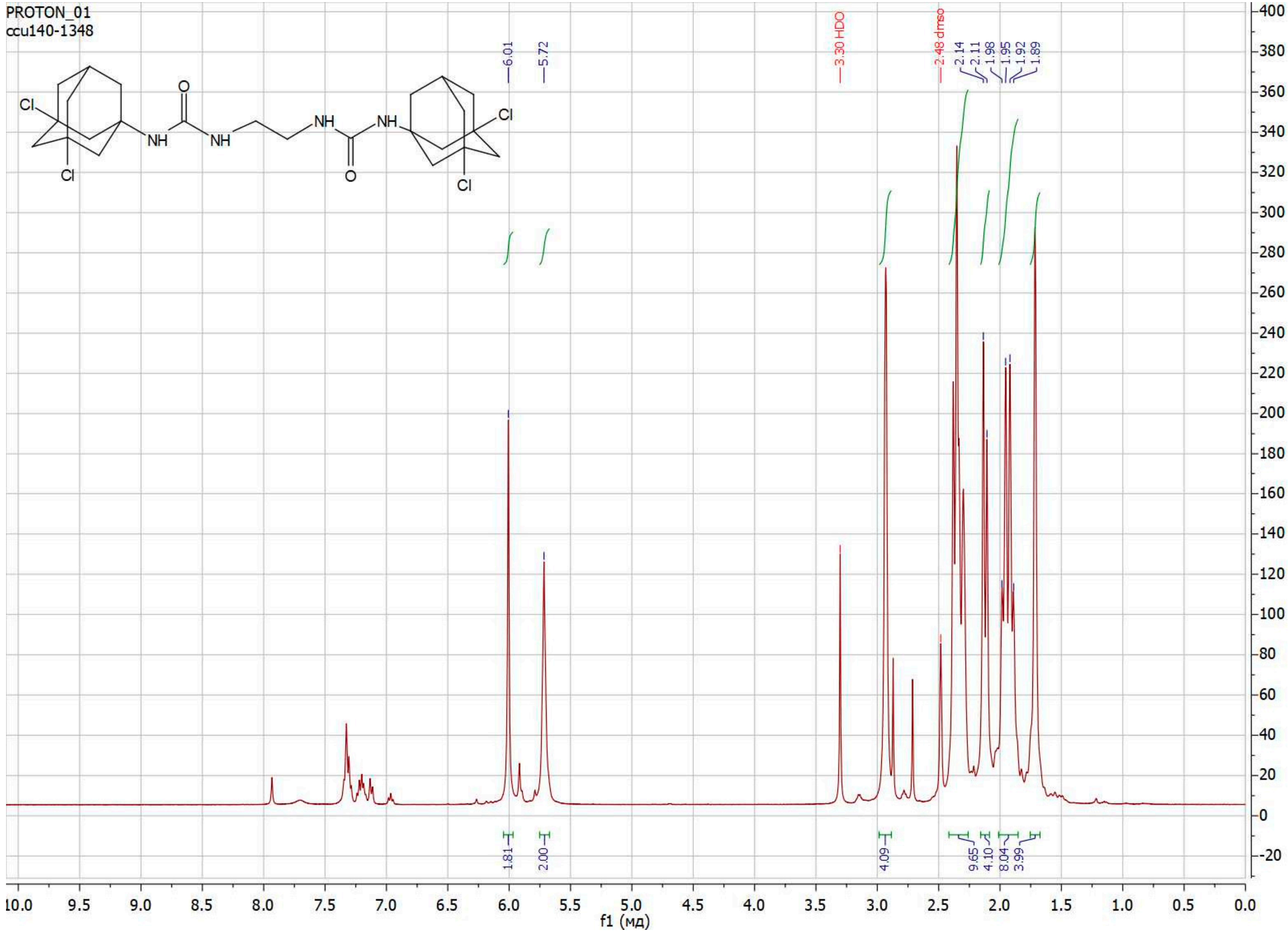

Figure S10. ^{13}C NMR of compound **7a**

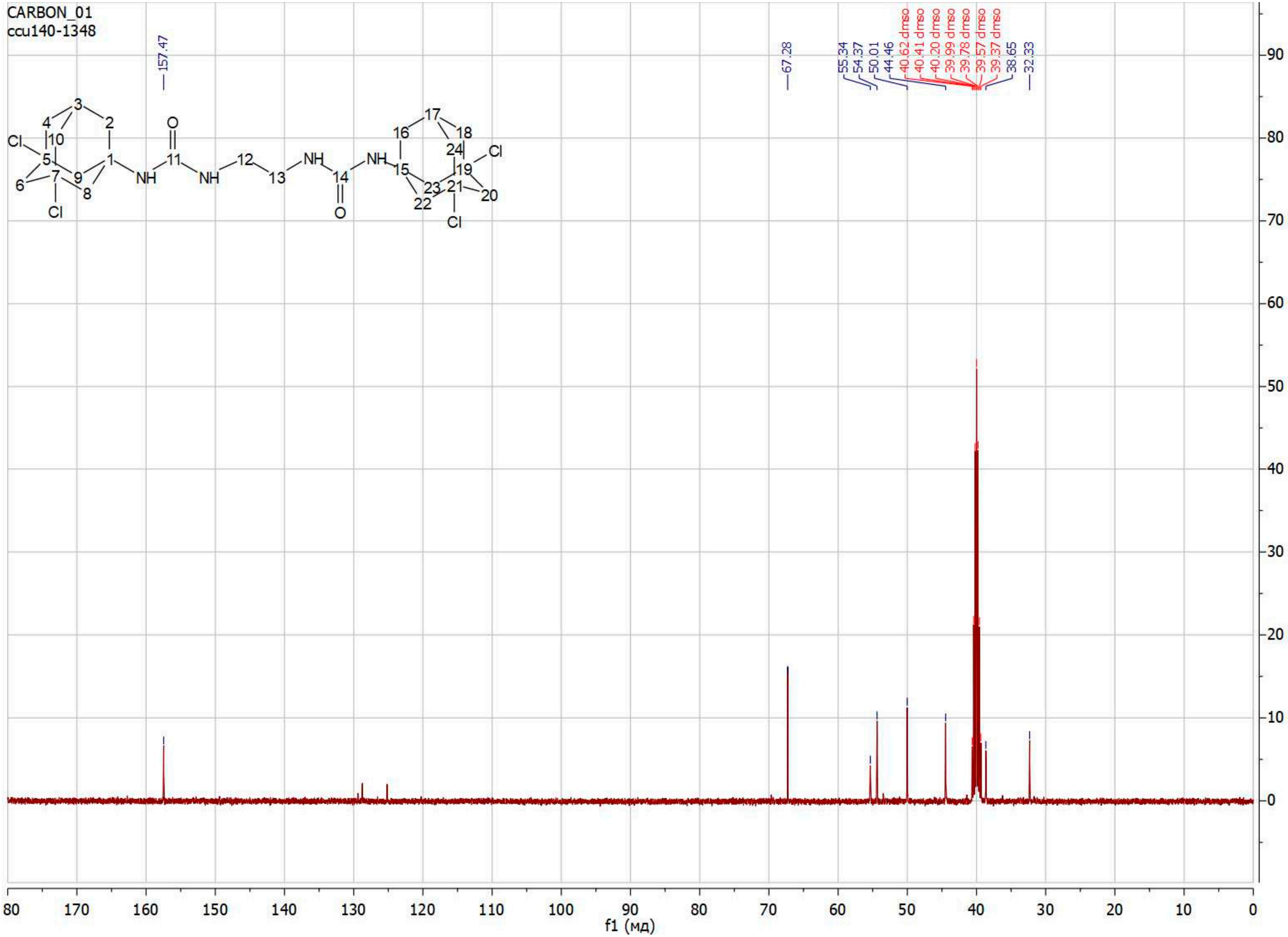

Figure S11. ^{1}H NMR of compound **7b**

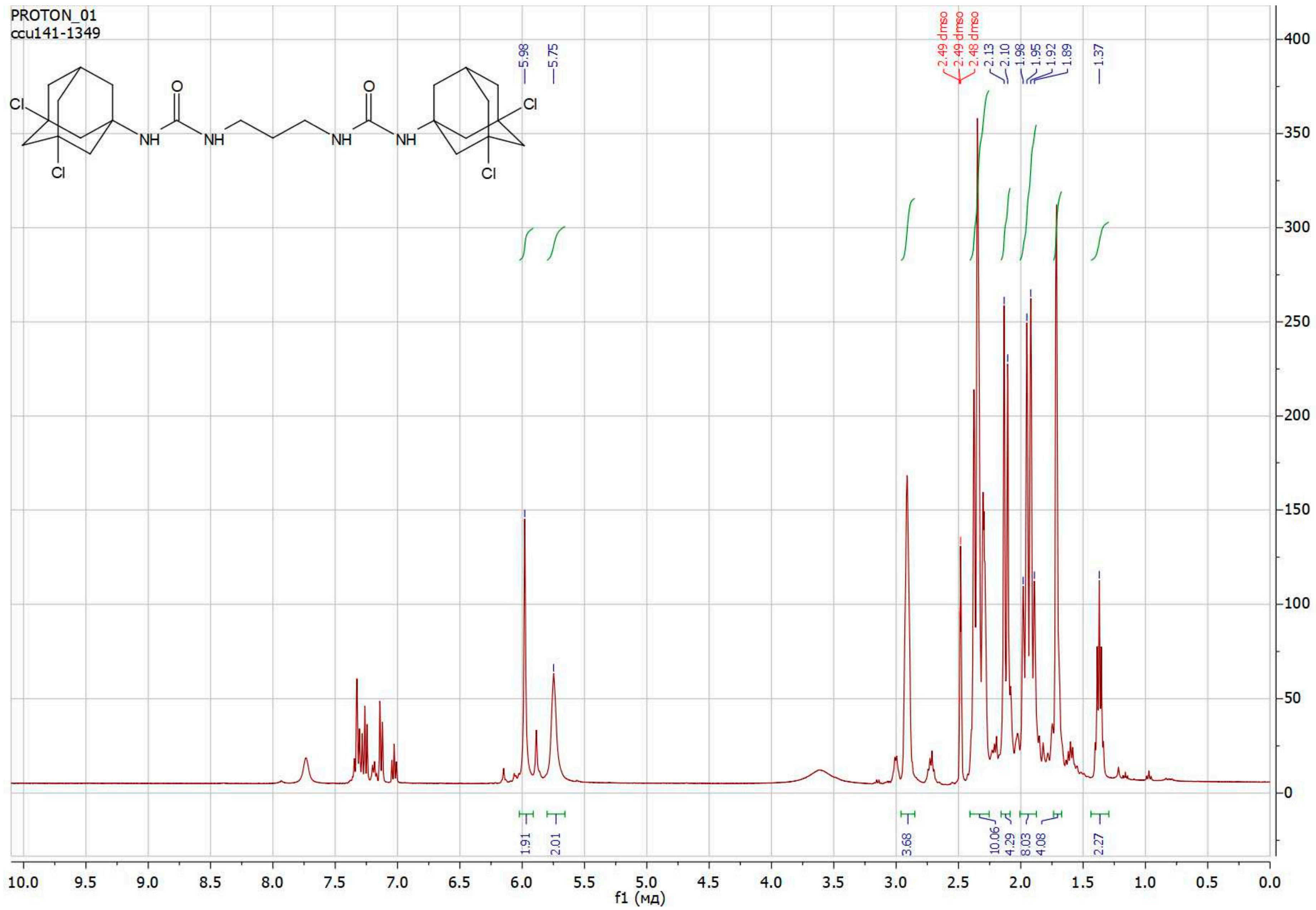

Figure S12. ^{13}C NMR of compound **7b**

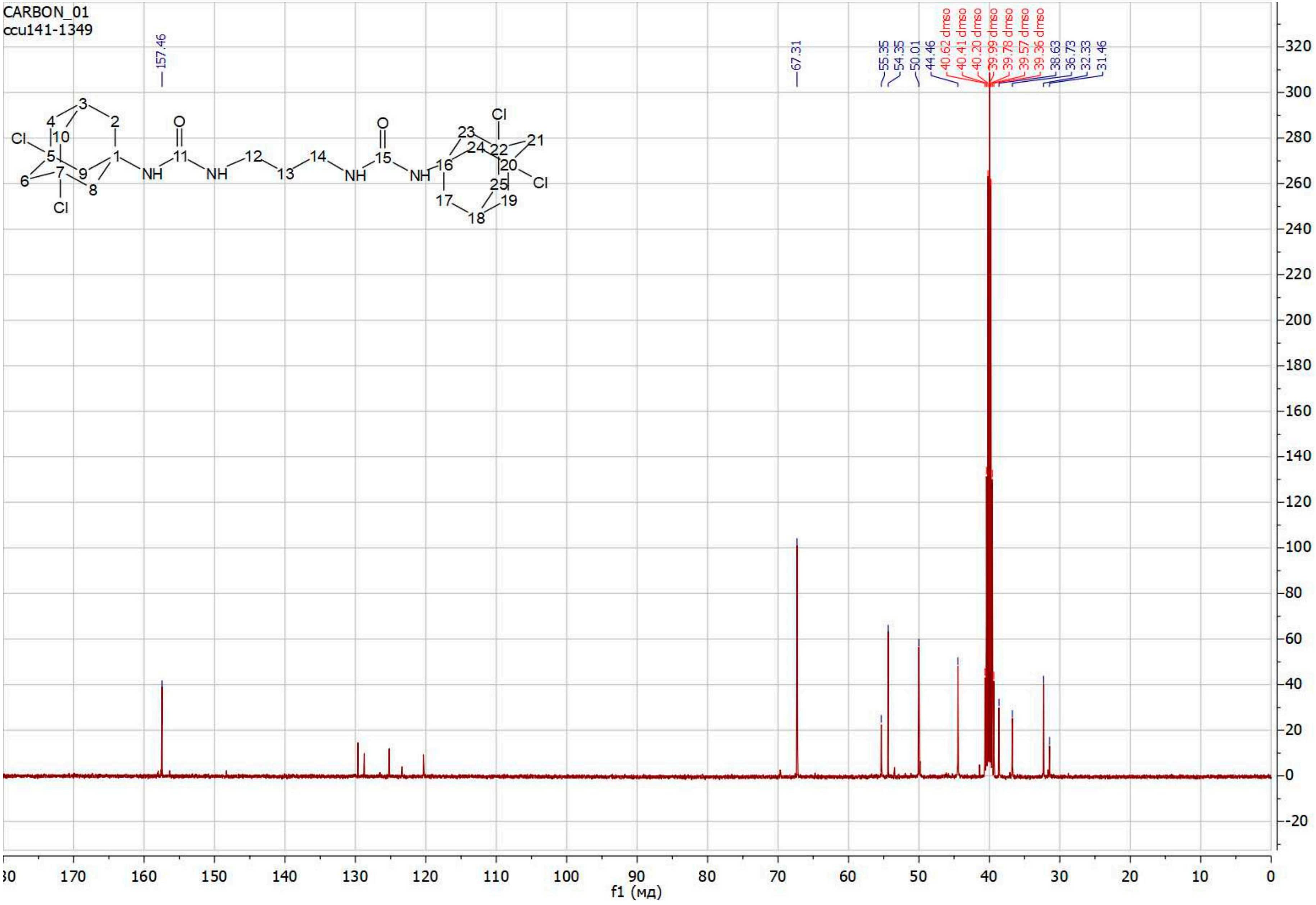

Figure S13. ^{1}H NMR of compound **7c**

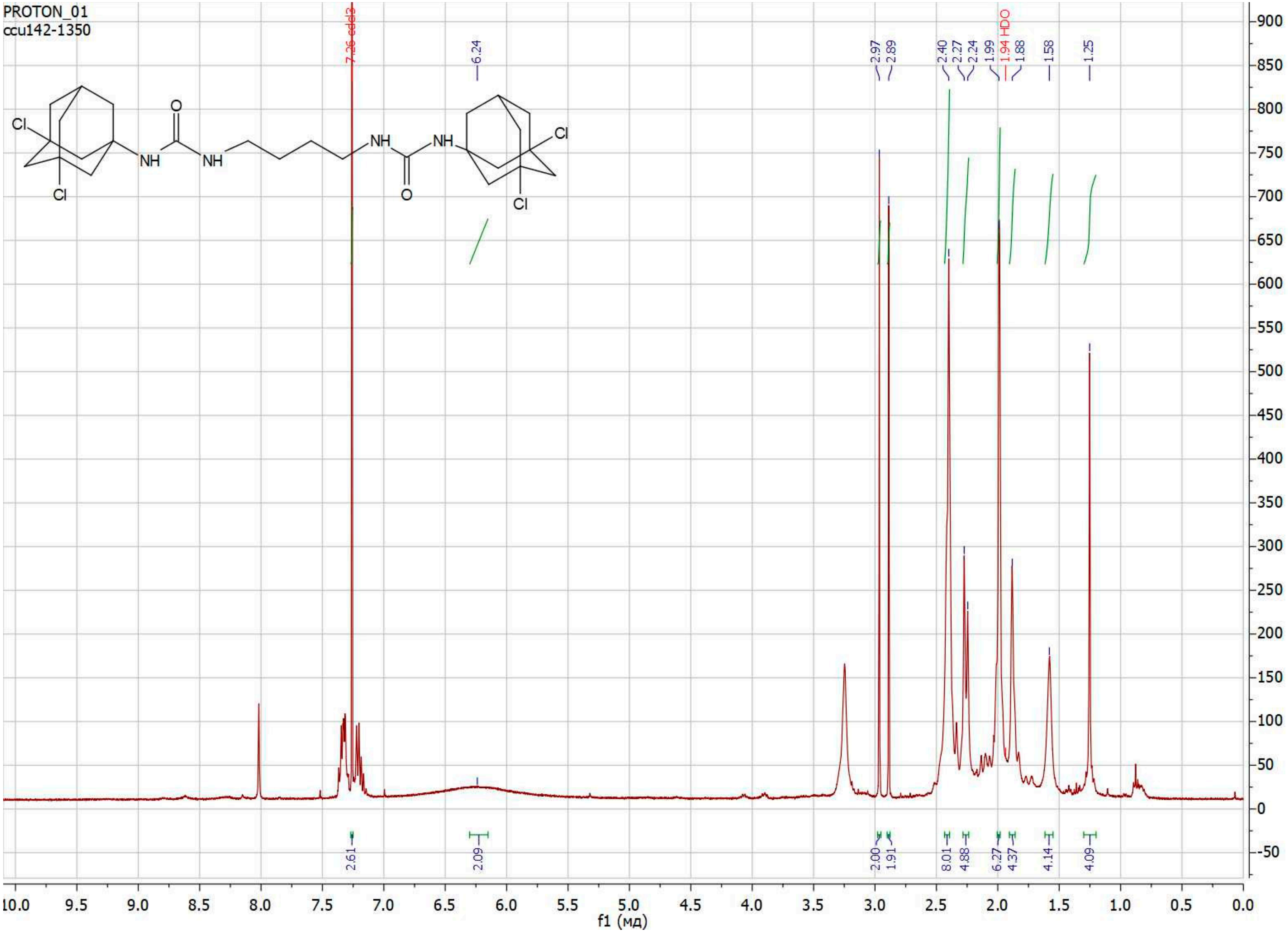

Figure S14. ^{13}C NMR of compound **7c**

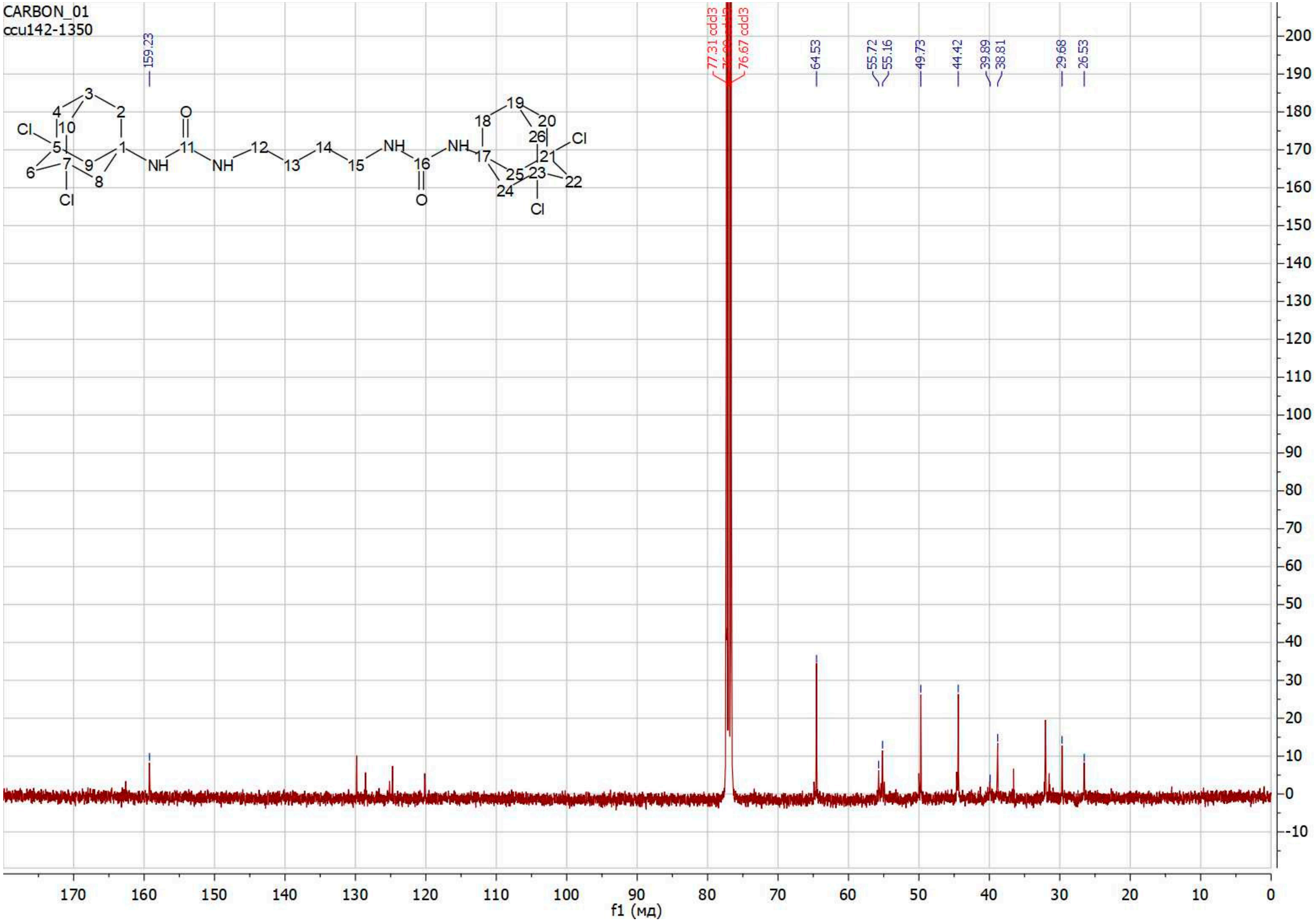

Figure S15. ^{1}H NMR of compound **7d**

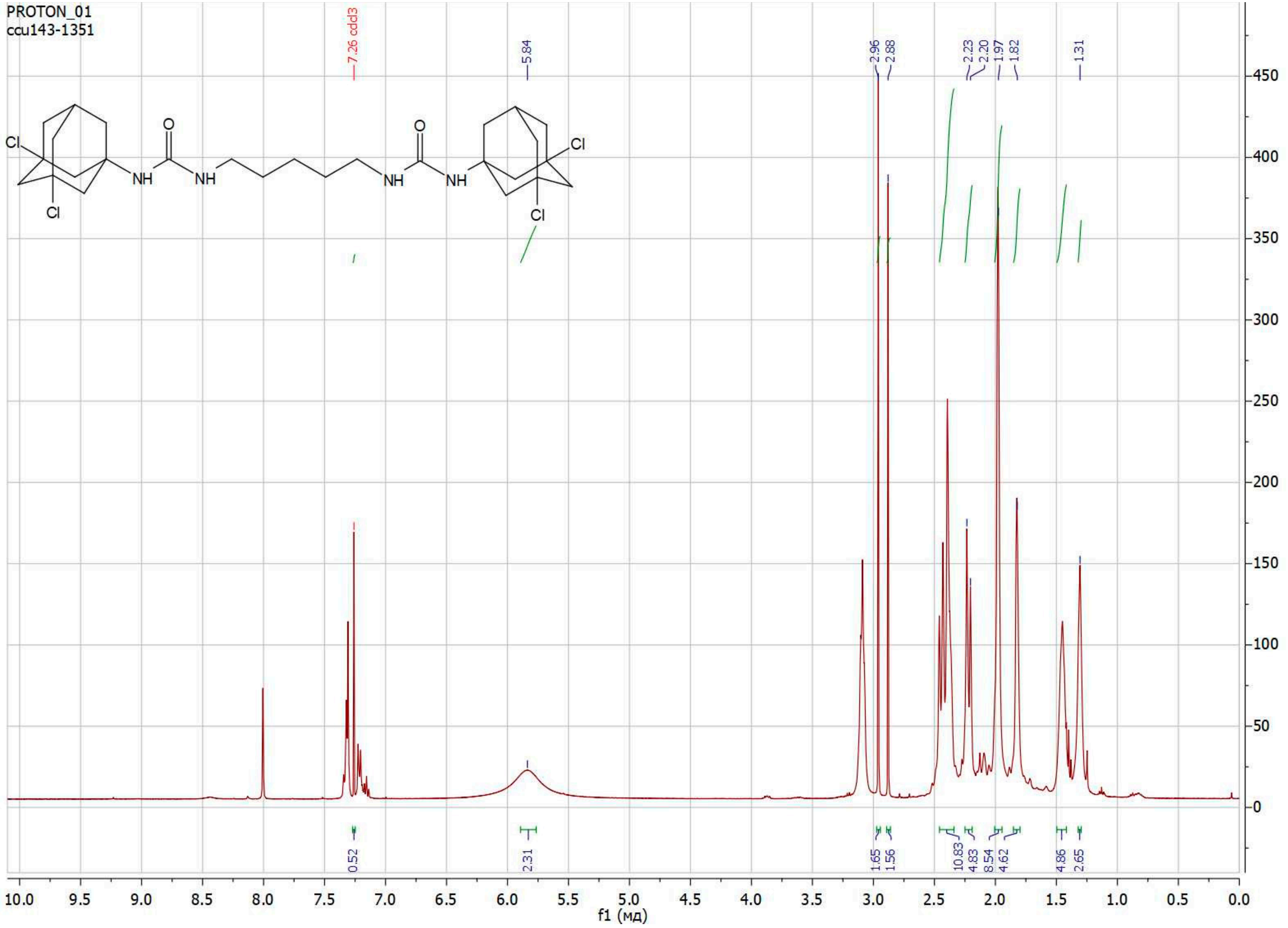

Figure S16. ^{13}C NMR of compound **7d**

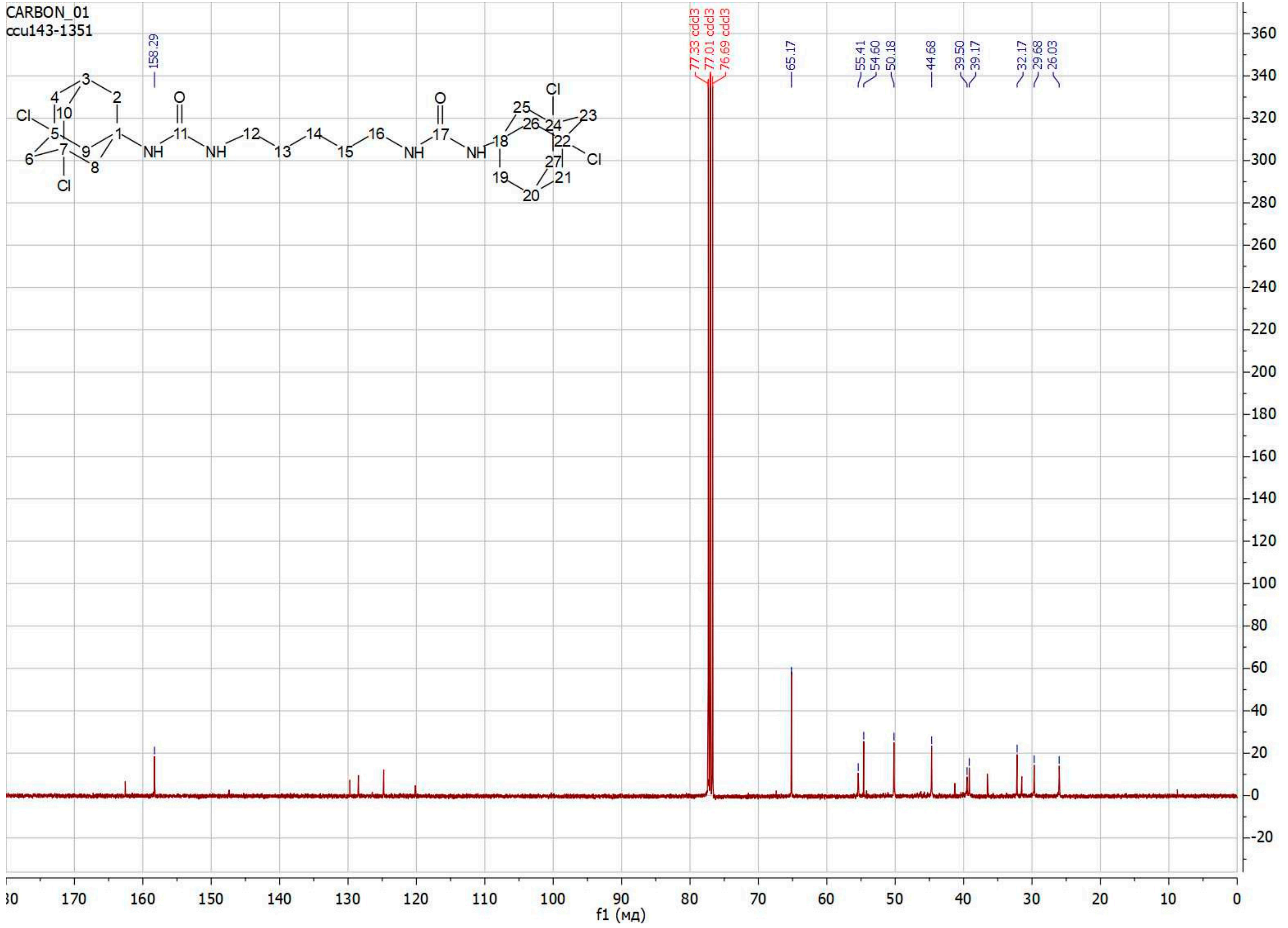

Figure S17. ^{1}H NMR of compound **7e**

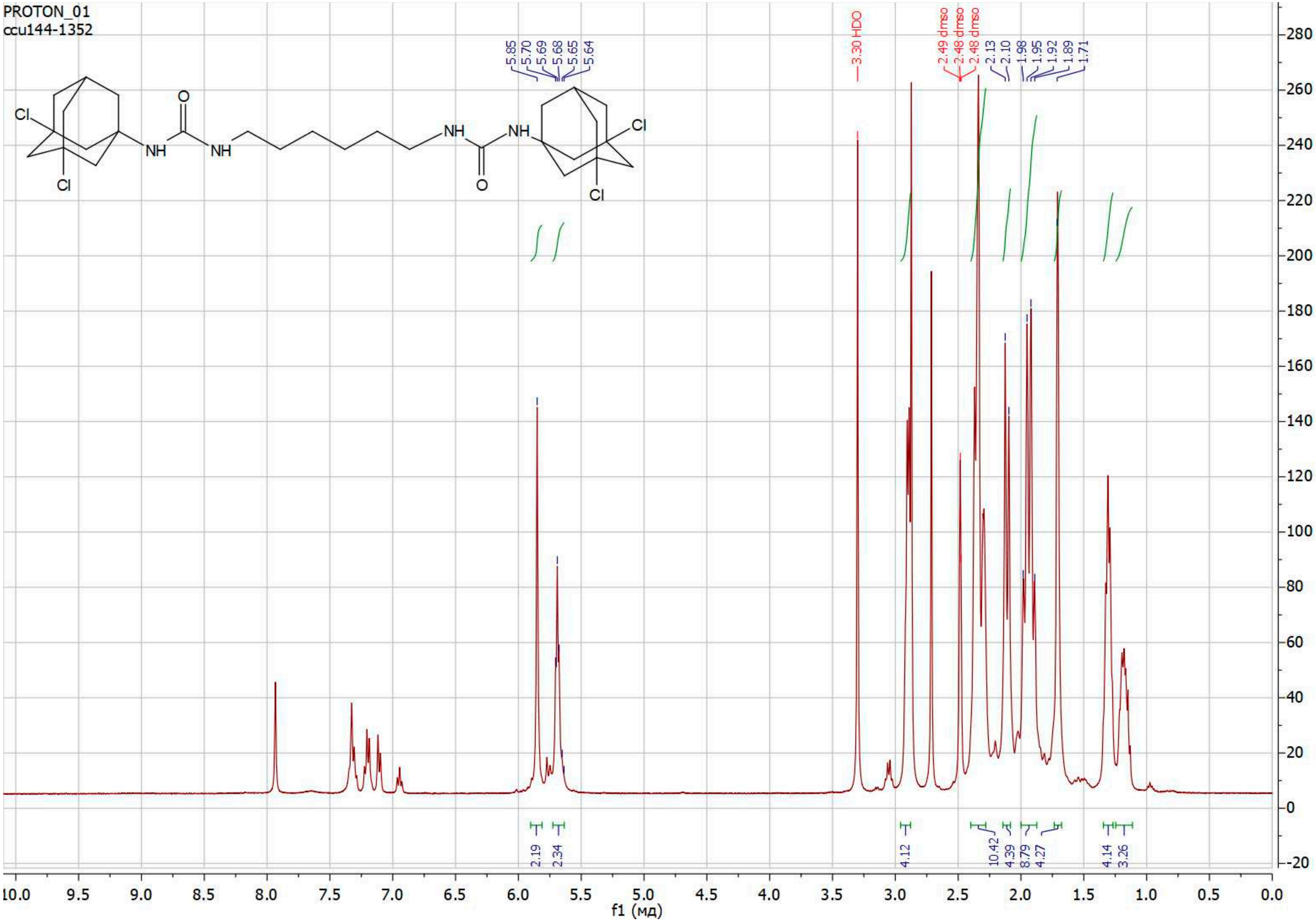

Figure S18. ^{13}C NMR of compound **7e**

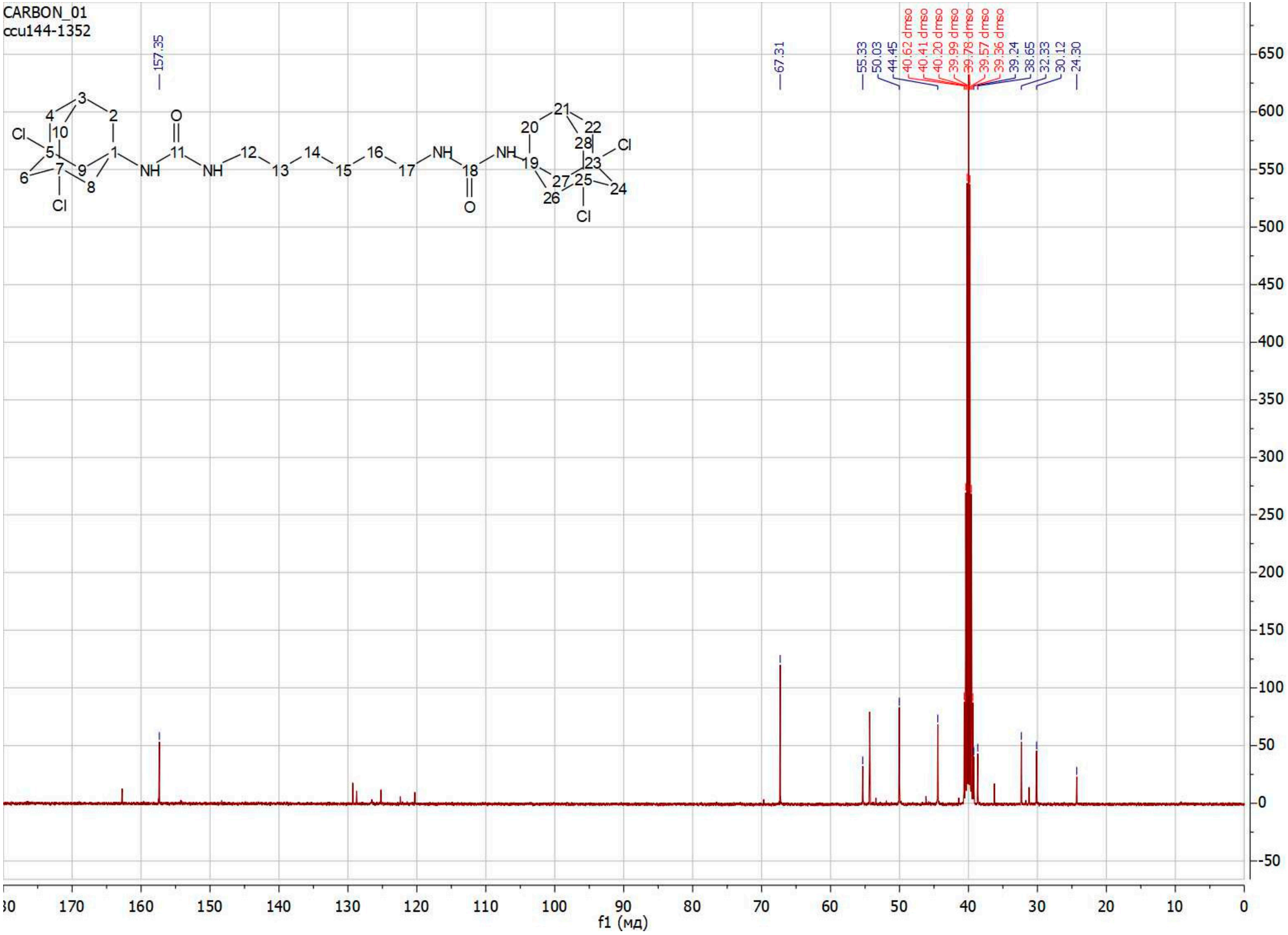

Figure S19. ^{1}H NMR of compound **7f**

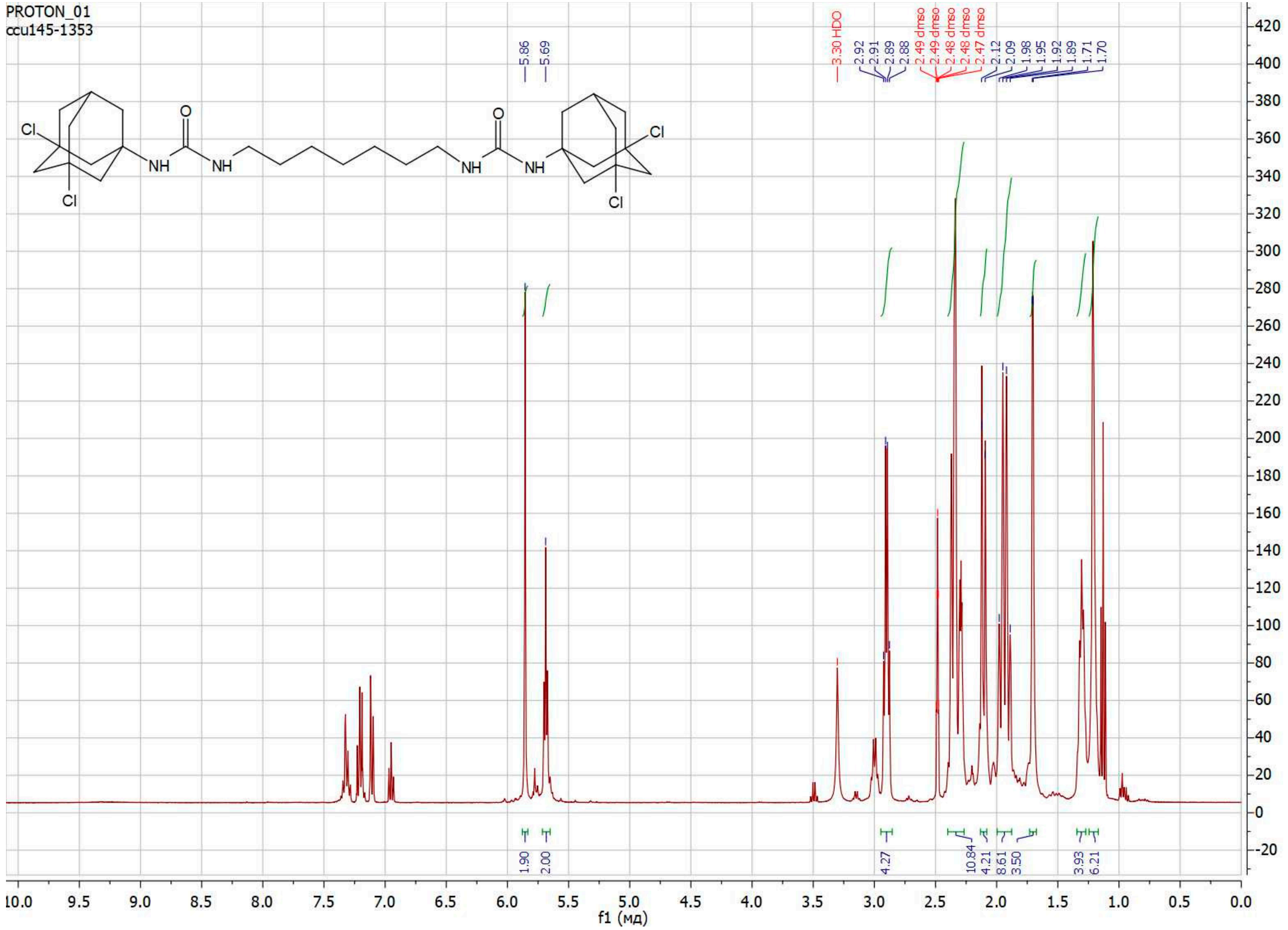

Figure S20. ^{13}C NMR of compound **7f**

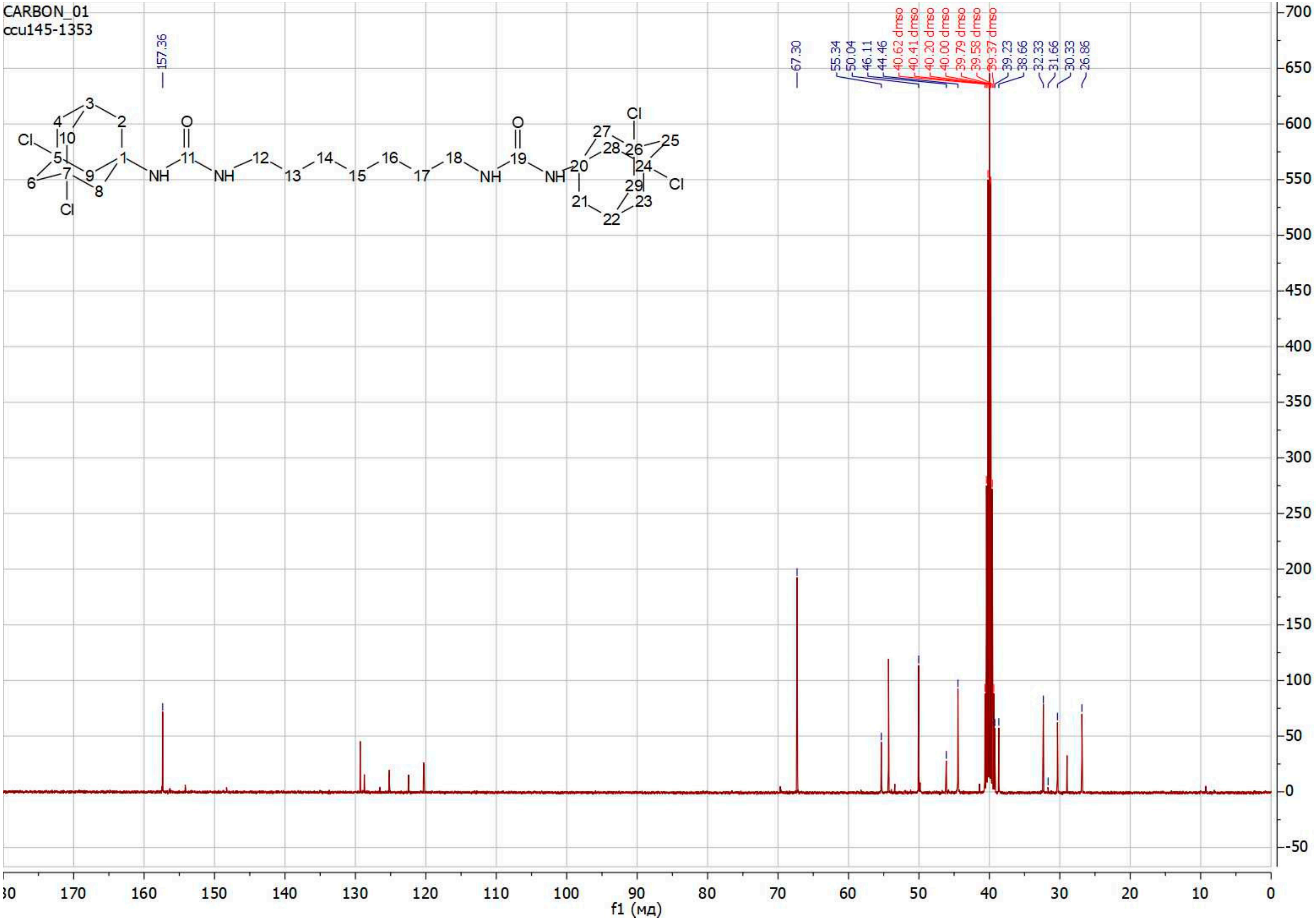

Figure S21. ^{1}H NMR of compound **7g**

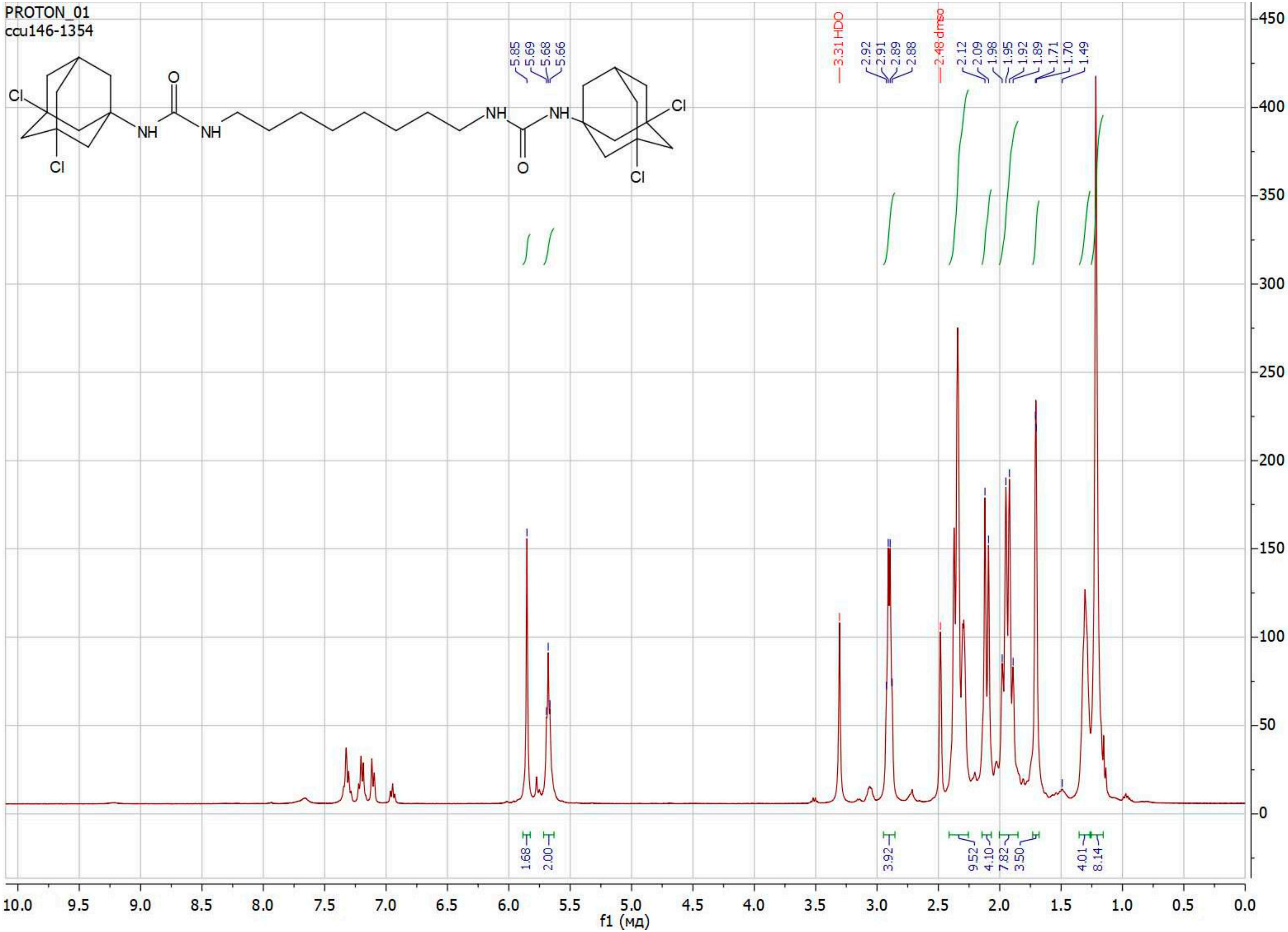

Figure S22. ^{13}C NMR of compound **7g**

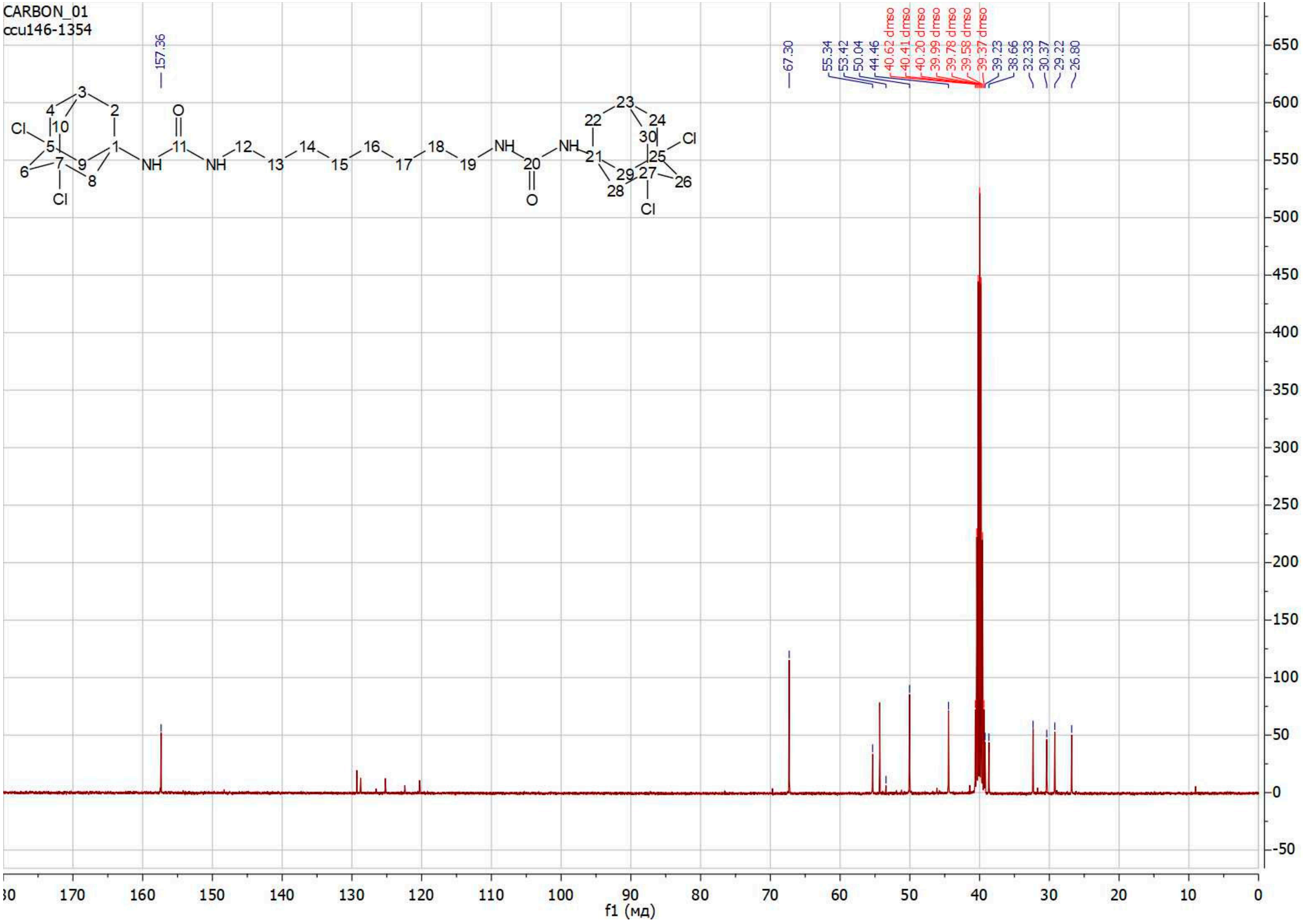

Figure S23. ^{1}H NMR of compound **7h**

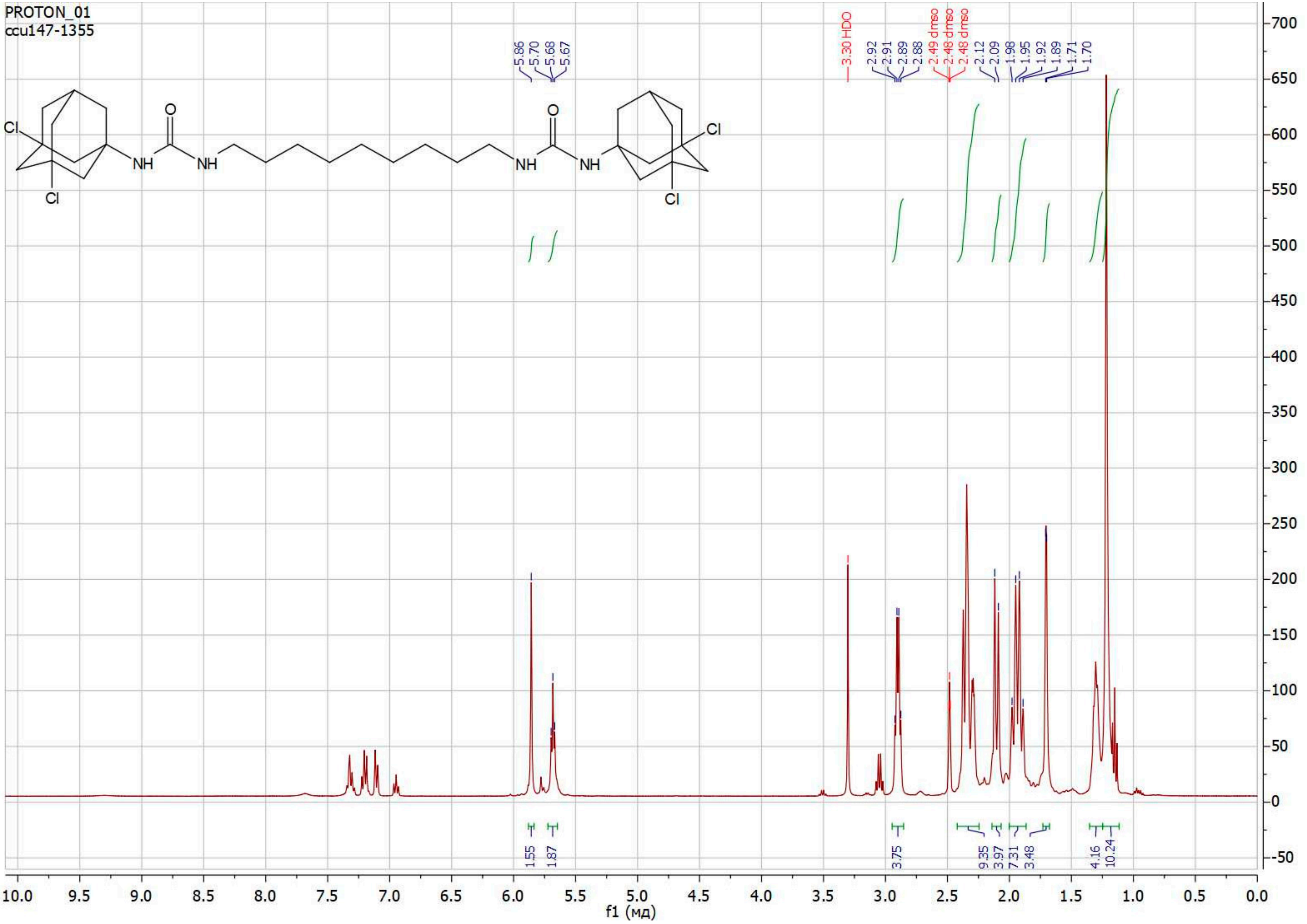

Figure S24. ^{13}C NMR of compound **7h**

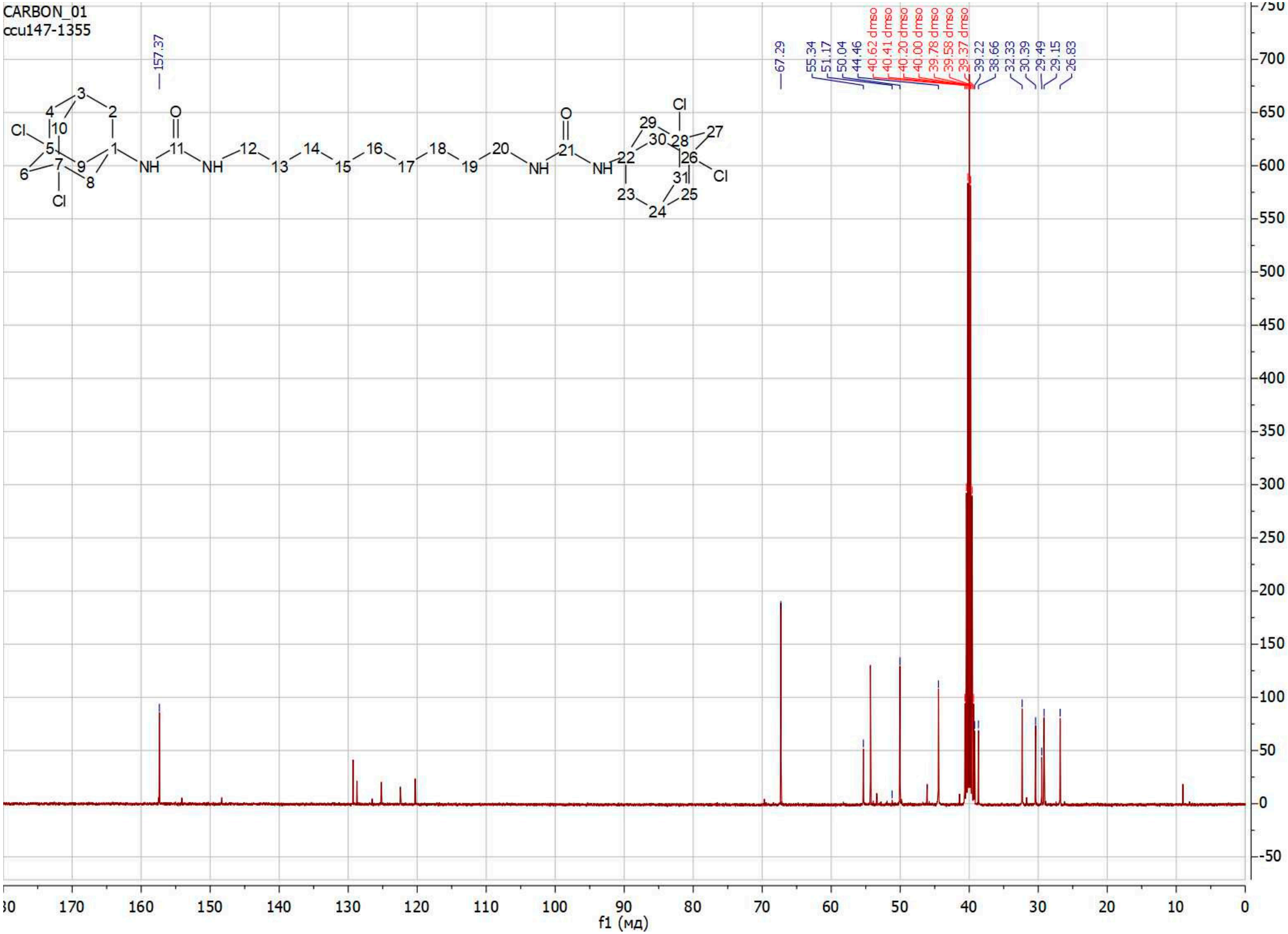

Figure S25. ^{1}H NMR of compound **7i**

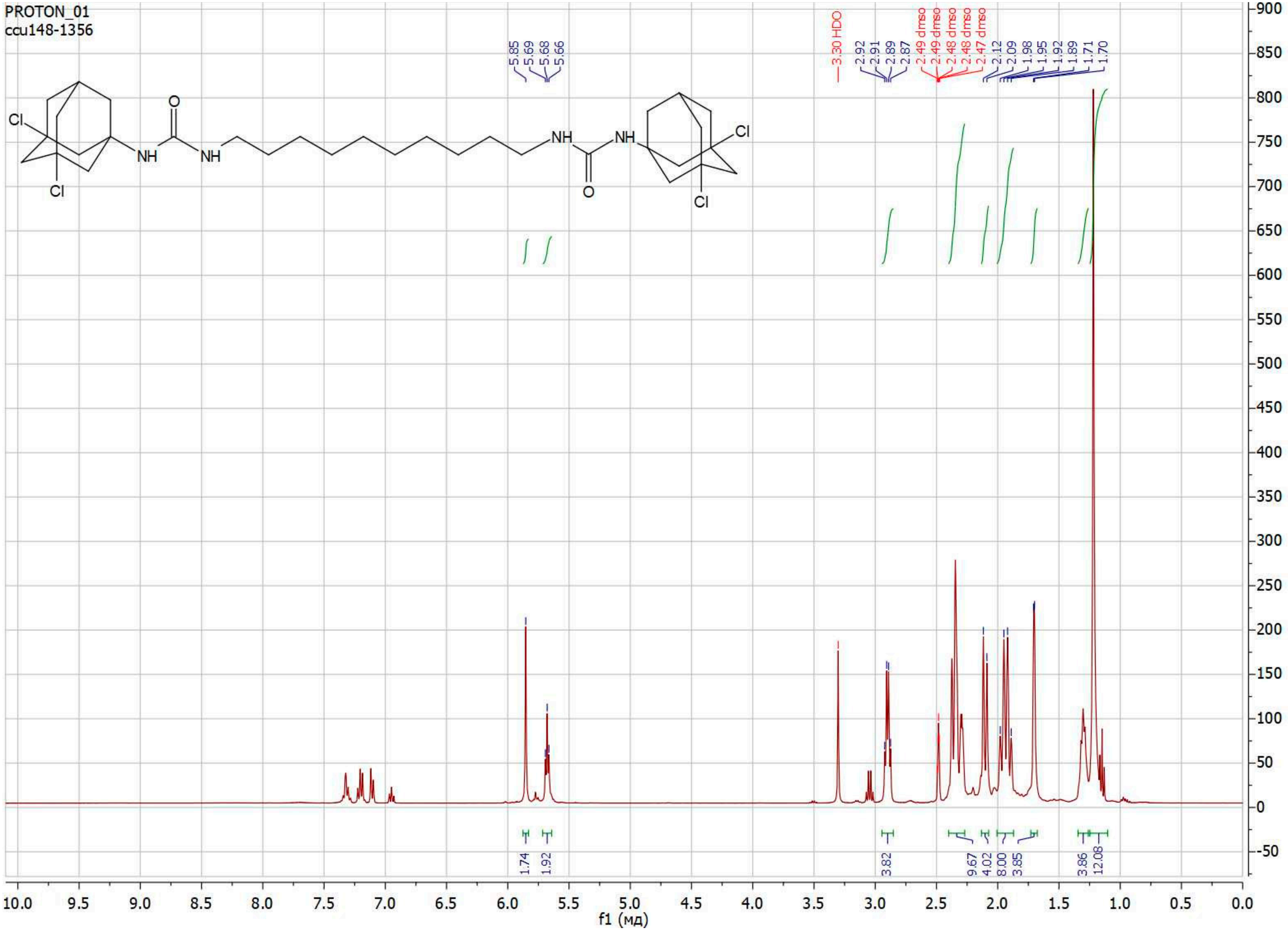

Figure S26. ^{13}C NMR of compound **7i**

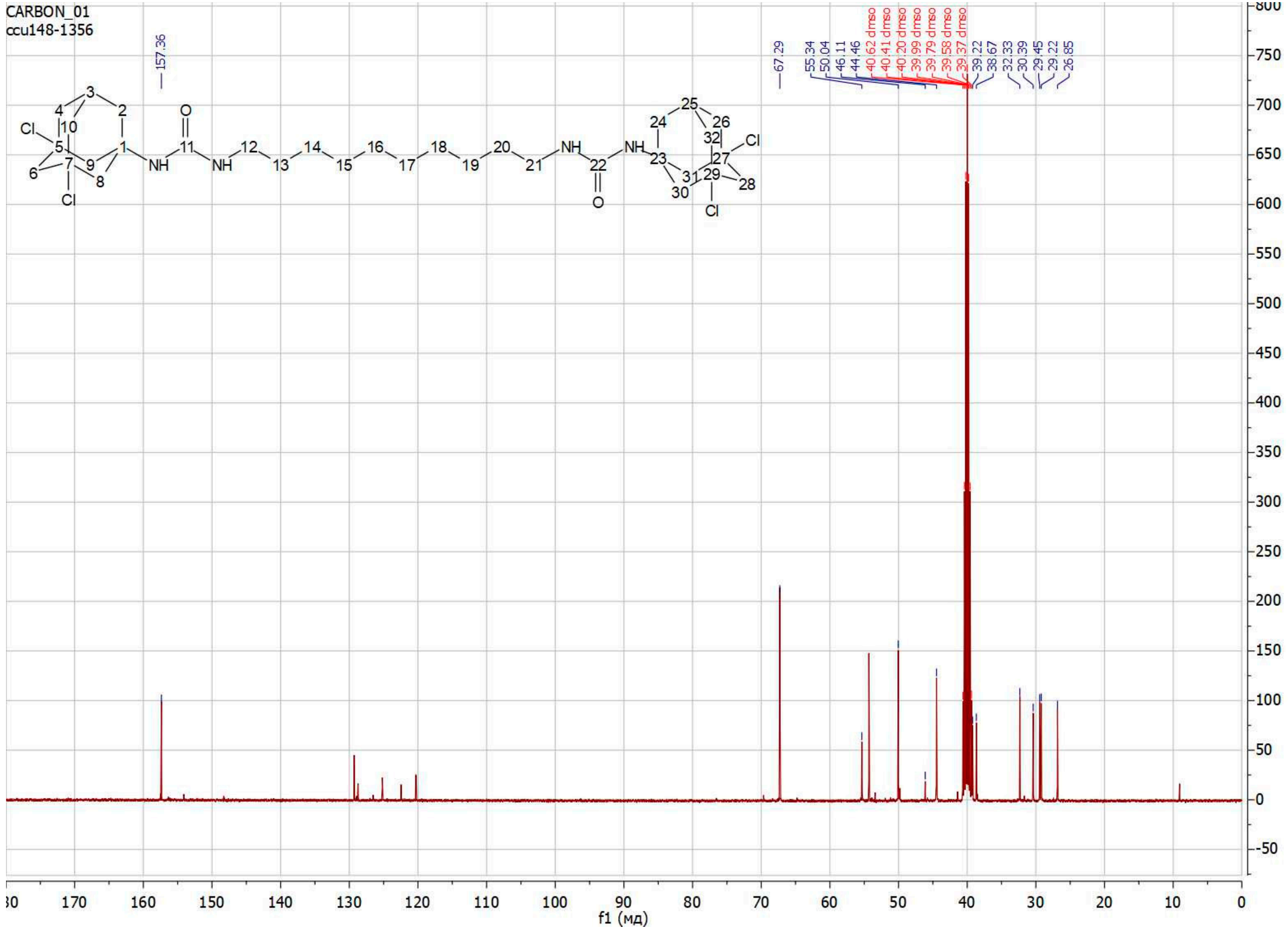

Figure S27. ^{1}H NMR of compound **9**

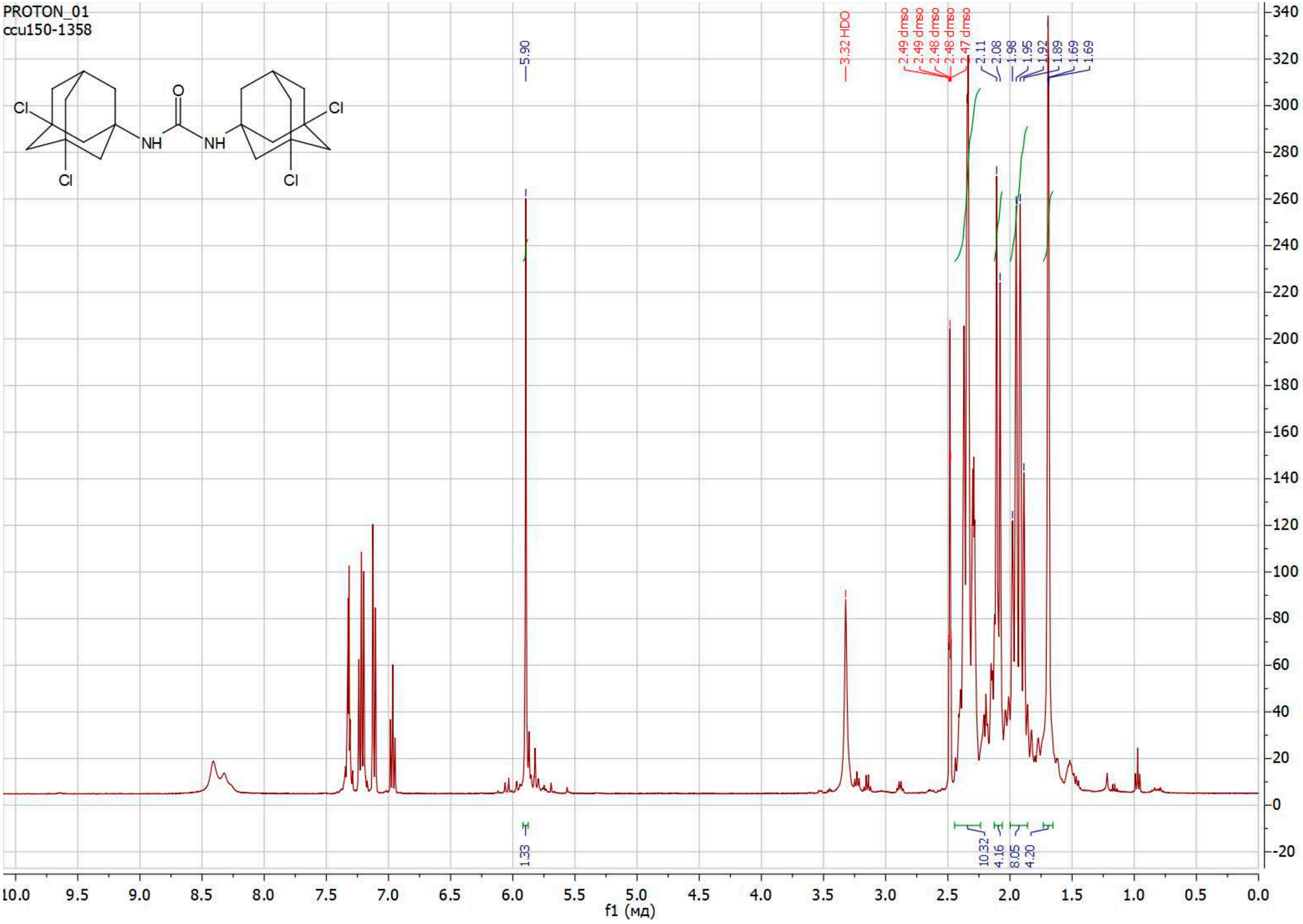

Figure S28. ^{13}C NMR of compound **9**

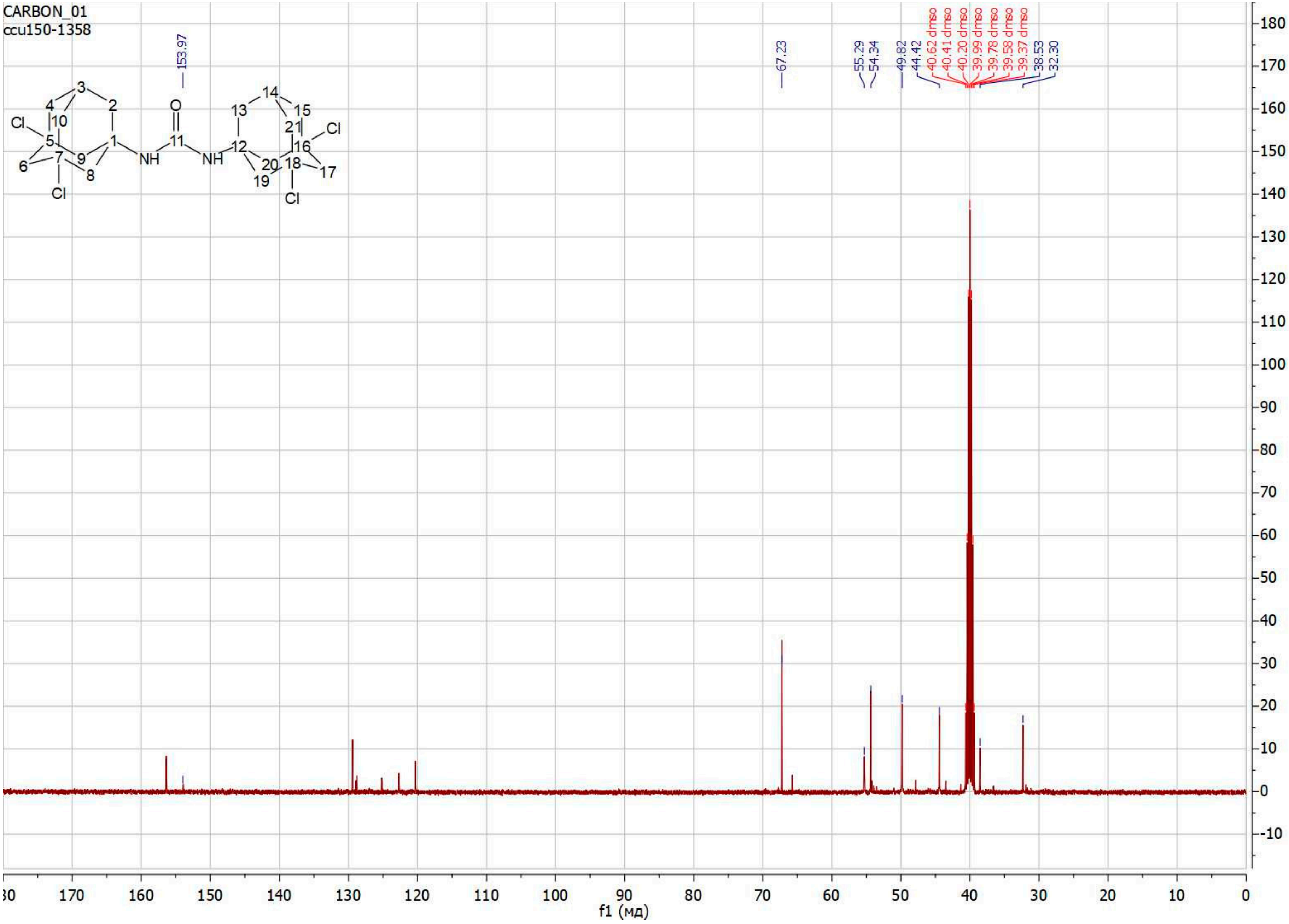

Figure S29. ^{1}H NMR of compound **11**

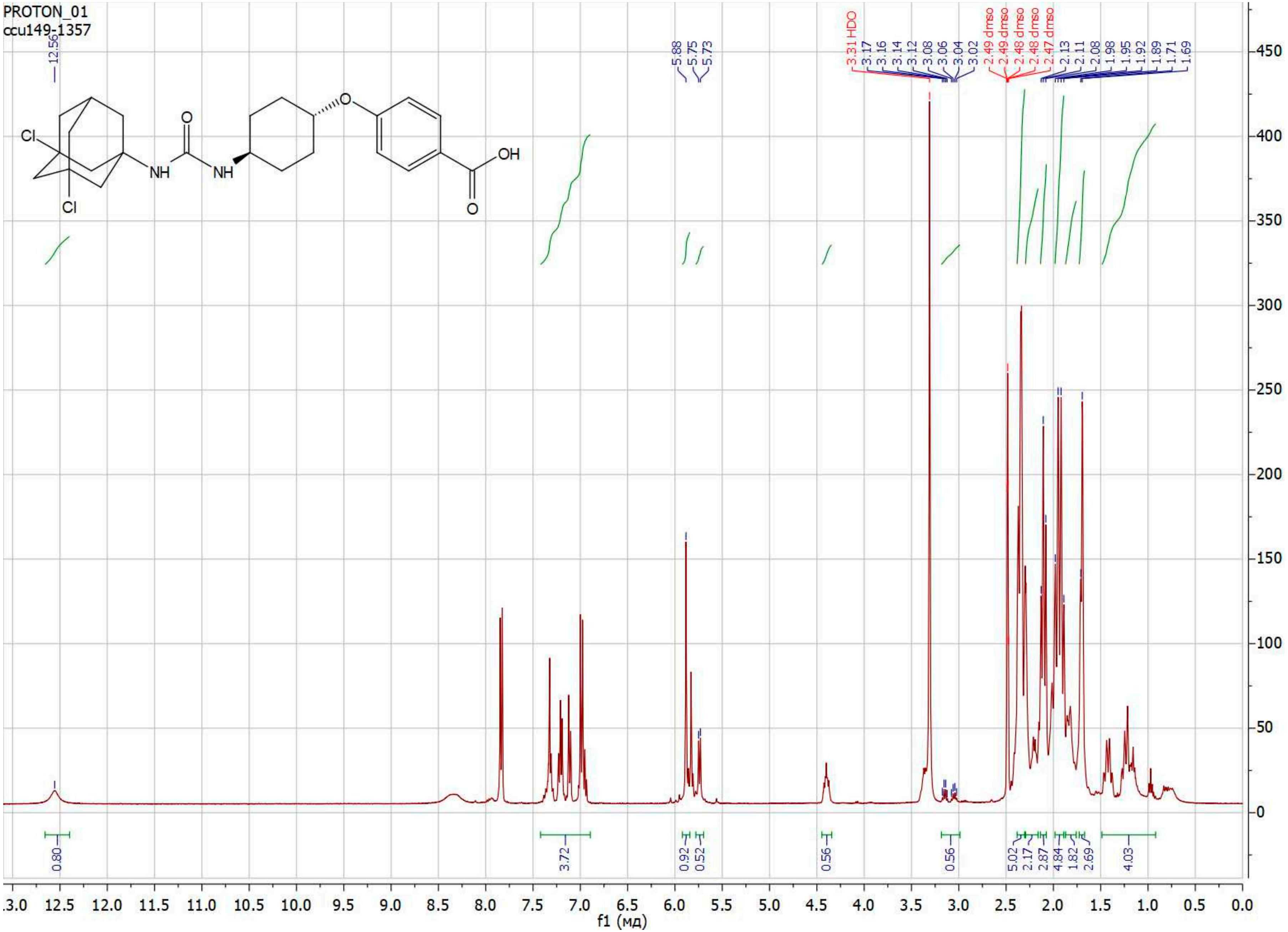

Figure S30. ^{13}C NMR of compound **11**

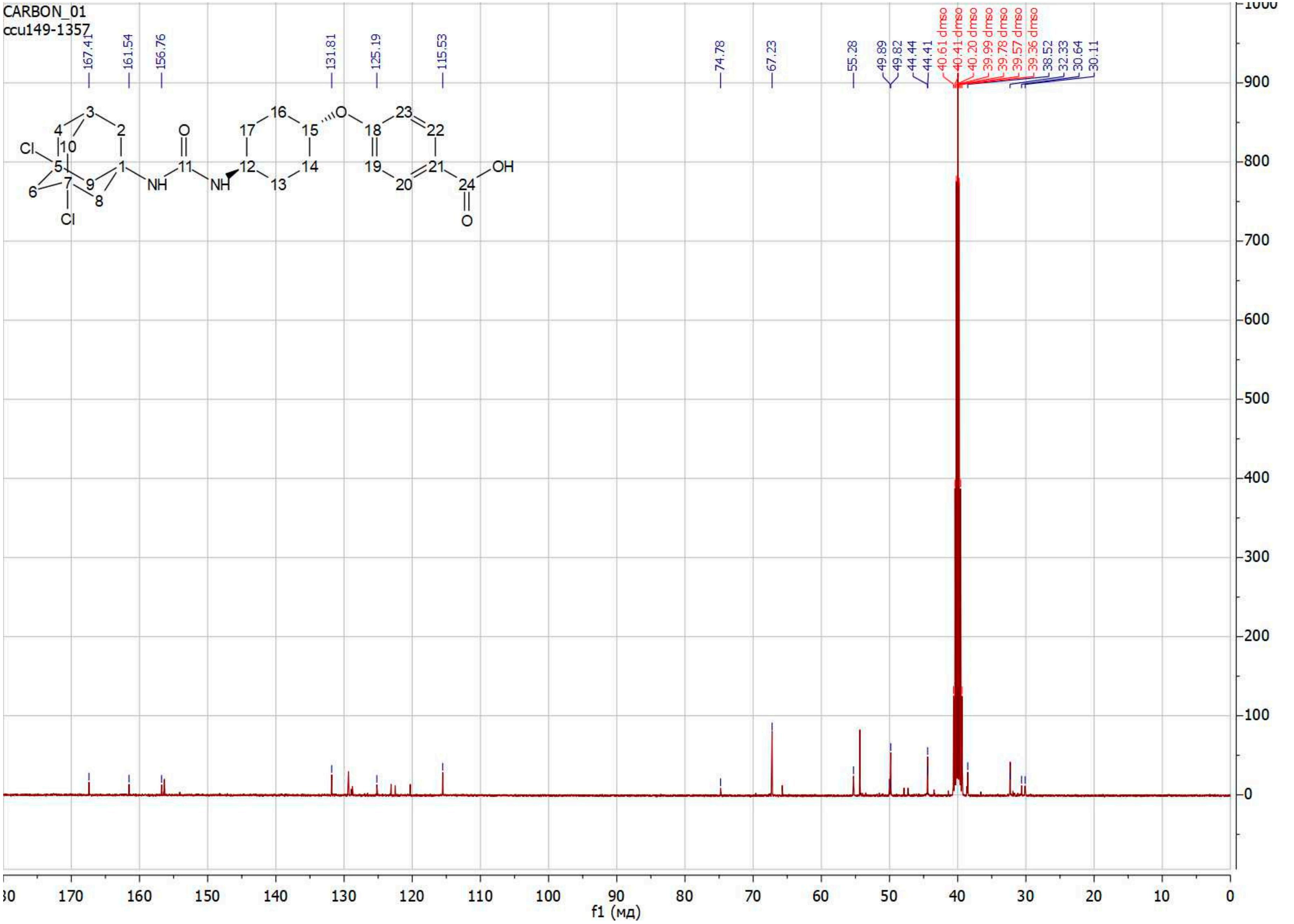

Supplement: Supplementary file 1 [file ijms-25-00338-s001.zip › ijms-2764360-supplementary.pdf]
